# Targeting gliovascular connexins prevents inflammatory blood-brain barrier leakage and astrogliosis

**DOI:** 10.1172/jci.insight.135263

**Published:** 2022-08-22

**Authors:** Marijke De Bock, Maarten De Smet, Stijn Verwaerde, Hanane Tahiri, Steffi Schumacher, Valérie Van Haver, Katja Witschas, Christian Steinhäuser, Nathalie Rouach, Roosmarijn E. Vandenbroucke, Luc Leybaert

**Affiliations:** 1 Physiology Group, Department of Basic and Applied Medical Sciences, Faculty of Medicine and Health Sciences, Ghent University, Ghent, Belgium.; 2 Institute of Cellular Neurosciences, Faculty of Medicine, University of Bonn, Bonn, Germany.; 3 Center for Interdisciplinary Research in Biology, College de France, CNRS, INSERM, Université PSL, Paris, France.; 4 Department of Biomedical Molecular Biology, Faculty of Sciences, Ghent University, Ghent, Belgium.; 5 Inflammation Research Center, VIB, Ghent, Belgium.

**Keywords:** Inflammation, Neuroscience, Calcium, Endothelial cells, Ion channels

## Abstract

The blood-brain barrier is formed by capillary endothelial cells expressing connexin 37 (Cx37), Cx40, and Cx43 and is joined by closely apposed astrocytes expressing Cx43 and Cx30. We investigated whether connexin-targeting peptides could limit barrier leakage triggered by LPS-induced systemic inflammation in mice. Intraperitoneal LPS administration increased endothelial and astrocytic Cx43 expression; elevated TNF-α, IL-1β, IFN-γ, and IL-6 in plasma and IL-6 in the brain; and induced barrier leakage recorded over 24 hours. Barrier leakage was largely prevented by global Cx43 knockdown and Cx43/Cx30 double knockout in astrocytes, slightly diminished by endothelial Cx43 knockout, and not protected by global Cx30 knockout. Intravenous administration of Gap27 or Tat-Gap19 peptides just before LPS also prevented barrier leakage, and intravenously administered BAPTA-AM to chelate intracellular calcium was equally effective. Patch-clamp experiments demonstrated LPS-induced Cx43 hemichannel opening in endothelial cells, which was suppressed by Gap27, Gap19, and BAPTA. LPS additionally triggered astrogliosis that was prevented by intravenous Tat-Gap19 or BAPTA-AM. Cortically applied Tat-Gap19 or BAPTA-AM to primarily target astrocytes also strongly diminished barrier leakage. In vivo dye uptake and in vitro patch-clamp showed Cx43 hemichannel opening in astrocytes that was induced by IL-6 in a calcium-dependent manner. We conclude that targeting endothelial and astrocytic connexins is a powerful approach to limit barrier failure and astrogliosis.

## Graphical abstract

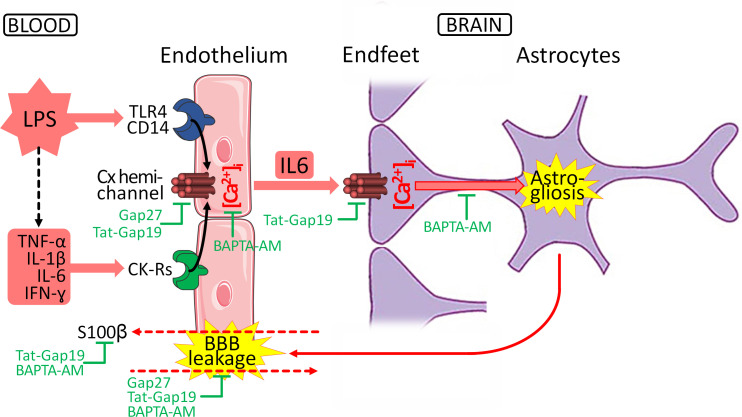

## Introduction

Efficient neuronal signaling in the central nervous system (CNS) strictly depends on a balanced and well-controlled microenvironment around glial cells, synapses, and axons. This is achieved by a series of physical barriers interposed between the nervous tissue, the blood, and the CSF, which collectively protect the brain from fluctuations in blood and CSF composition. The most stringent barrier is the blood-brain barrier (BBB) that is formed by an extremely dense network of capillary endothelial cells that separate the brain from the blood in such a way that almost every neuron has a local barrier interface in its microenvironment. Unique features of brain capillary endothelial cells (BCECs) are responsible for the BBB’s restrictive function. These include a complex belt of tight junctions and adherens junctions sealing off the paracellular cleft, the presence of a thick and continuous glycocalyx, severely reduced nonspecific vesicular activity, and a strictly regulated set of transporters that controls nearly all passage into and out of the brain. Endothelial barrier properties are furthermore controlled by surrounding partner cells, including astrocytes and pericytes (1). Among these, astrocytes have been best characterized for their influence on BBB function. Their endfeet projections nearly completely surround the capillary endothelium and exert a trophic influence on the BBB that maintains the expression of junctional proteins, transporters, and other barrier features in brain capillaries (2). Astrocytes respond to all forms of brain injury and disease through astrogliosis, thereby producing neurotrophic factors, growth factors, cytokines, chemokines, neurotransmitters, reactive oxygen species, and proteases that may detrimentally affect BBB function (2–4). Astrocytic endfeet are closely appositioned to capillary endothelial cells but separated from the latter by a basal lamina. Several connexins are expressed at this gliovascular interface, including endothelial connexin 37 (Cx37), Cx40, and Cx43 and astrocytic Cx43 and Cx30 (5, 6), that have established channel roles in each cell type but do not interconnect astrocytes with endothelial cells by gap junctions (7). Astrocytic endfeet are known to play a role in the communication of calcium (Ca^2+^) signals alongside the vessel wall and in the process of neurovascular coupling (7–10); however, fairly little is known about their contribution to endothelial barrier function. Astrocytic deletion of Cx30 and Cx43 has no effect on baseline barrier permeability but renders it more vulnerable to increased hydrostatic pressure (6). Moreover, absence of astrocytic Cx43 promotes endothelial immune activation, allowing the infiltration of lymphocytes, macrophages, and neutrophils (11). Here, we investigated the contribution of endothelial and astrocytic Cx43 to BBB alterations induced by systemically administered LPS, a Gram-negative bacterial wall component that triggers an innate immune response through activation of Toll-like receptor 4 and CD14 (12–14). We used 2 approaches to target endothelial cells and astrocytes, by applying inhibitors of connexin channel function and intracellular Ca^2+^ dynamics either intravenously (IV) or directly at the exposed brain cortex where cell-permeating molecules have access to astrocytes via the glia limitans. We found that IV administered, barrier-impermeable peptide Gap27 strongly protected against barrier leakage, while barrier-permeable peptide Tat-Gap19 protected against barrier leakage and astrogliosis. Tat-Gap19 also strongly inhibited barrier leakage when applied to the cortical surface. Overall, barrier protection was not clearly linked to astrogliosis or to LPS-induced alterations in proinflammatory cytokines in blood and brain. Instead, barrier protection was linked to inhibition of Ca^2+^-dependent Cx43 hemichannel activation in endothelial cells and astrocytes, with brain IL-6 functioning as a hemichannel activator in astrocytes.

## Results

### Intraperitoneal injection of LPS induces systemic and cerebral inflammation along with barrier leakage.

**Figure 1:**
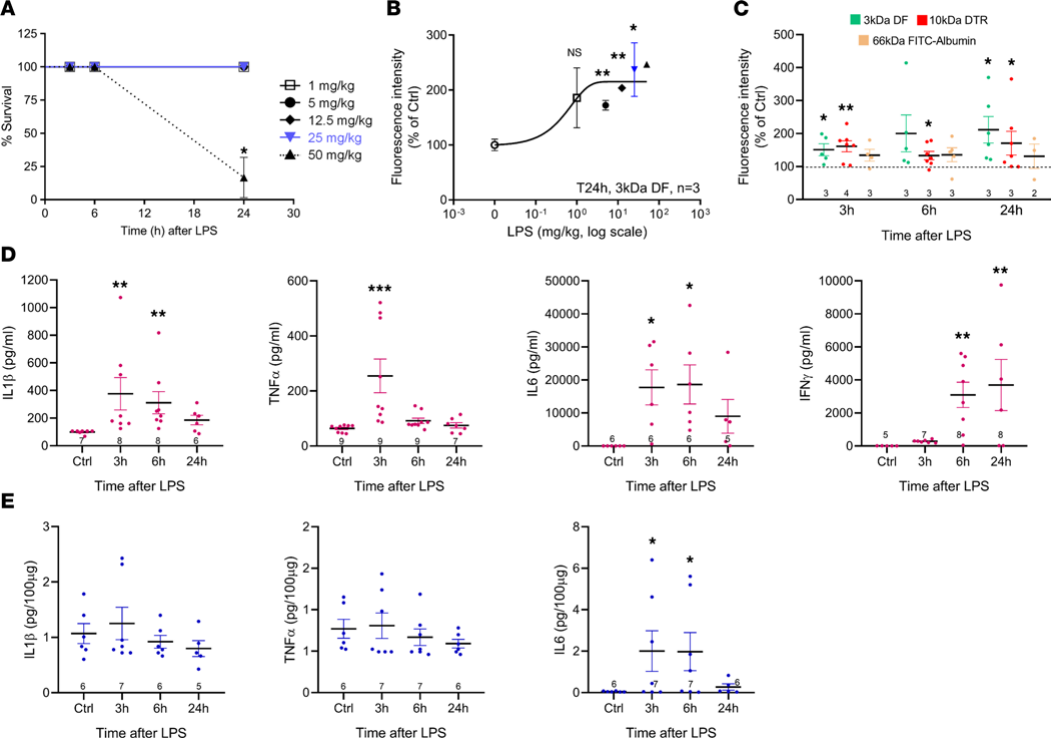
LPS-induced BBB leakage and inflammation in mice. (**A**) Kaplan-Meier plot illustrating survival for increasing doses of IP injected LPS. With the exception of the highest dose (50 mg/kg), LPS did not affect survival (*n* = 3 for 1, 5, 12.5, and 25 mg/kg and *n* = 6 for 50 mg/kg; for the 1–25 mg/kg dose range, survival is 100% and data points therefore overlap). (**B**) Dose-response curve for BBB leakage of 3 kDa dextran fluorescein (DF) (IV 30 mg/kg), 24 hours post-LPS. Leakage increased with increasing LPS dose, reaching a plateau at 25–50 mg/kg. (Mean ± SEM with *n* = 3 for all concentrations, except for 50 mg/kg, where *n* = 1 due to high mortality; Dunnett test comparison with no LPS). Symbols correspond to **A**. The 25 mg/kg dose was used in all further experiments (marked in blue). (**C**) LPS-induced BBB leakage at 3, 6, and 24 hours post-LPS, determined with 3 kDa DF (30 mg/kg), 10 kDa dextran Texas red (DTR; 100 mg/kg), and FITC-albumin (66 kDa, 660 mg/kg). Stars compare with Ctrl (saline IP; 1-sample *t* test). Numbers in the bars indicate experiments on different animals. (**D** and **E**) Plasma and brain levels of IL-1β, TNF-α, IFN-γ, and IL-6 following LPS. In plasma (**D**), all tested cytokines were significantly elevated; IFN-γ showed a delayed response. In the brain (**E**), only IL-6 increased with a time course as in plasma; IL-1β and TNF-α were not increased, and IFN-γ was not detectable. Stars indicate significant difference with Ctrl (saline IP; 1-way ANOVA, Dunnett test except for IL-1β and TNF-α, where nonparametric Kruskal-Wallis testing was used). *P < 0.05, **P < 0.01, and ***P < 0.001.

We tested various LPS doses (1–50 mg/kg intraperitoneally, IP) and found 100% mouse survival up to 25 mg/kg in the 24-hour experimental observation window (Figure 1A). Next, we verified the effect of the various LPS doses on barrier permeability, determined by the leakage of IV injected 3 kDa dextran-fluorescein (DF) into the brain (30 mg/kg, 10 minutes prior to sacrifice and brain isolation). Twenty-four hours after LPS, significant tracer leakage in the cerebral cortex was observed with 5 mg/kg LPS or higher, approaching a plateau at 25 mg/kg (Figure 1B). We chose the 25 mg/kg submaximal concentration for optimal signal stability compared with half-maximal effect concentration where small concentration changes produce large changes in leakage effect. To document LPS-induced barrier leakage for a range of distinct molecular weight tracers, we included 10 kDa dextran Texas red (DTR) and 66 kDa FITC-albumin and found leakage for the 3 and 10 kDa dextran tracers but not for the 66 kDa tracer, indicating barrier function was still intact for albumin (Figure 1C). To characterize peripheral inflammation, we analyzed plasma levels of the proinflammatory cytokines IL-1β, TNF-α, IFN-γ, and IL-6 and found that all but IFN-γ were significantly increased at the earliest (3 hours) time point, followed by a decline toward the end of the recording (Figure 1D), as observed in rodents treated with lower LPS doses (15, 16). IFN-γ showed a delayed response and increased at 6 and 24 hours after LPS injection. In the brain, IL-1β and TNF-α levels did not significantly change over the 24-hour window, and IFN-γ levels fell below the assay’s detection limit, notwithstanding their strong peripheral levels. By contrast, brain IL-6 levels reliably followed the time course of plasma IL-6, peaking at 3 and 6 hours post-LPS and declining thereafter (Figure 1E).

### LPS triggers increased Cx43 expression in barrier endothelial cells and astrocytes.

**Figure 2:**
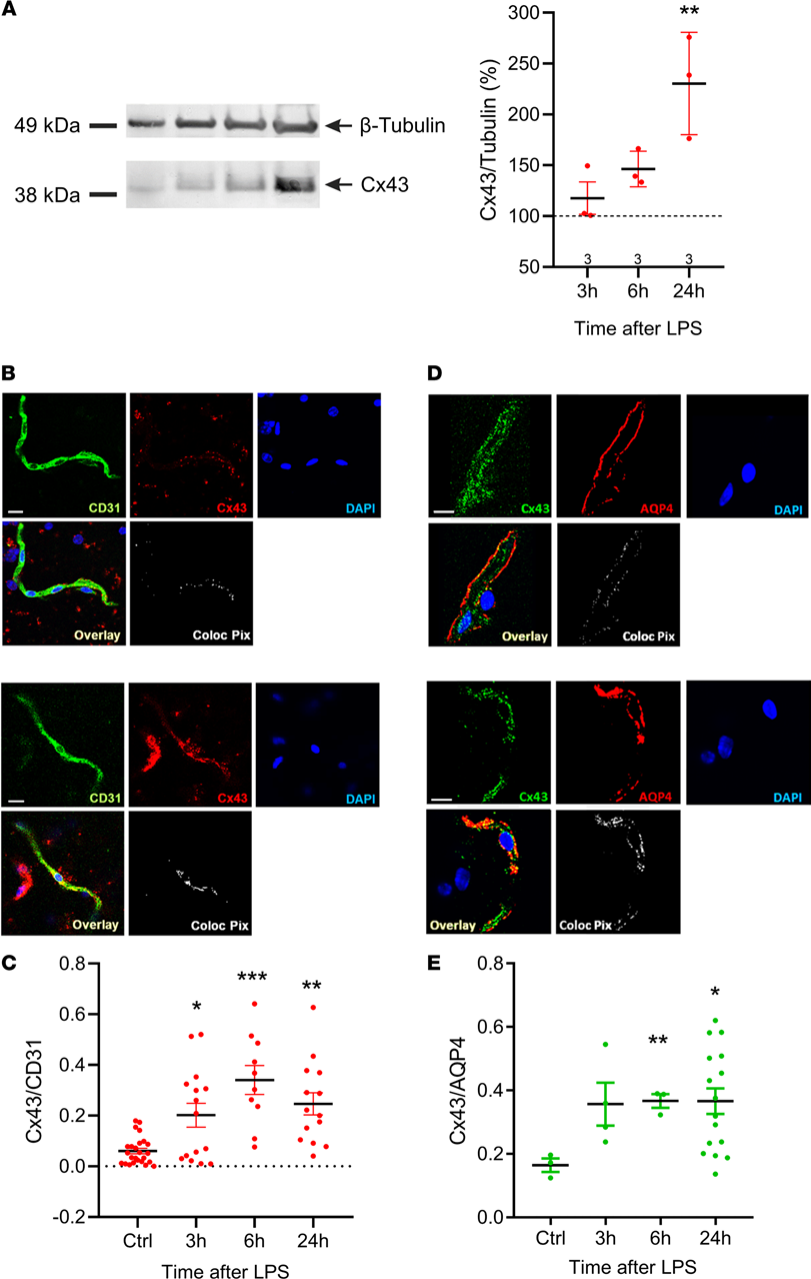
Cx43 expression increases after IP LPS treatment. (**A**) SDS-PAGE and Western blotting experiments showed a strong Cx43 increase in freshly isolated mouse brain capillary endothelial cells treated with LPS (1 μg/mL) (**P < 0.01, 1-sample *t* test). (**B**) Staining for the endothelial cell marker CD31 and Cx43 in isolated brain capillaries of Ctrl and LPS-injected mice (25 mg/kg). Pixels in white (Coloc Pix, size 180 nm^2^) illustrate combined Cx43/CD31 positivity. Scale bar: 10 μm. (**C**) Summary white Coloc Pix counts of experiments as in **B**, illustrating strongly increased Cx43 expression in brain capillary endothelial cells in animals that received LPS. (**D** and **E**) Colocalization analysis of Cx43 and the astrocytic endfeet marker AQP4 demonstrated increased Cx43 expression in endfeet remnants in capillaries of animals that received LPS. Scale bar: 10 μm. Stars in **C** and **E** indicate significant difference compared with normalized Ctrl (1-way ANOVA, Dunnett test). *P < 0.05, **P < 0.01, and ***P < 0.001.

Although the role of BBB endothelial Cx43 is far less characterized than it is for glial cells, accumulating evidence suggests its involvement in barrier leakage (5). In vivo, Cx43 expression is low in brain microvascular endothelium but increases after pathological insults such as ischemia, trauma, and cerebral cavernous malformations (17–19). Here, we found increased Cx43 expression in primary cultures of mouse BCECs exposed to LPS (1 μg/mL) in vitro (Figure 2A), as well as in intact brain capillaries isolated from mice treated with LPS (25 mg/kg) (Figure 2, B and C). Staining of isolated brain capillaries with the endothelial marker CD31 and the astrocytic endfeet marker AQP4 allowed us to distinguish Cx43 expression in these 2 cell types. Cx43 changes in astrocytic endfeet are shown in Figure 2, D and E, demonstrating a significant increase at 6 hours while significance was attained at 3 hours for capillary endothelial cells (Figure 2C). We also tested the effect of LPS treatment on endothelial Cx37 and Cx40 and found those to be lowered 24 hours after LPS administration, however, without attaining statistical significance (Supplemental Figure 1; supplemental material available online with this article; https://doi.org/10.1172/jci.insight.135263DS1).

### LPS-induced barrier leakage is inhibited by IV administered connexin-targeting peptides and intracellular Ca^2+^ chelator BAPTA-AM.

**Figure 3:**
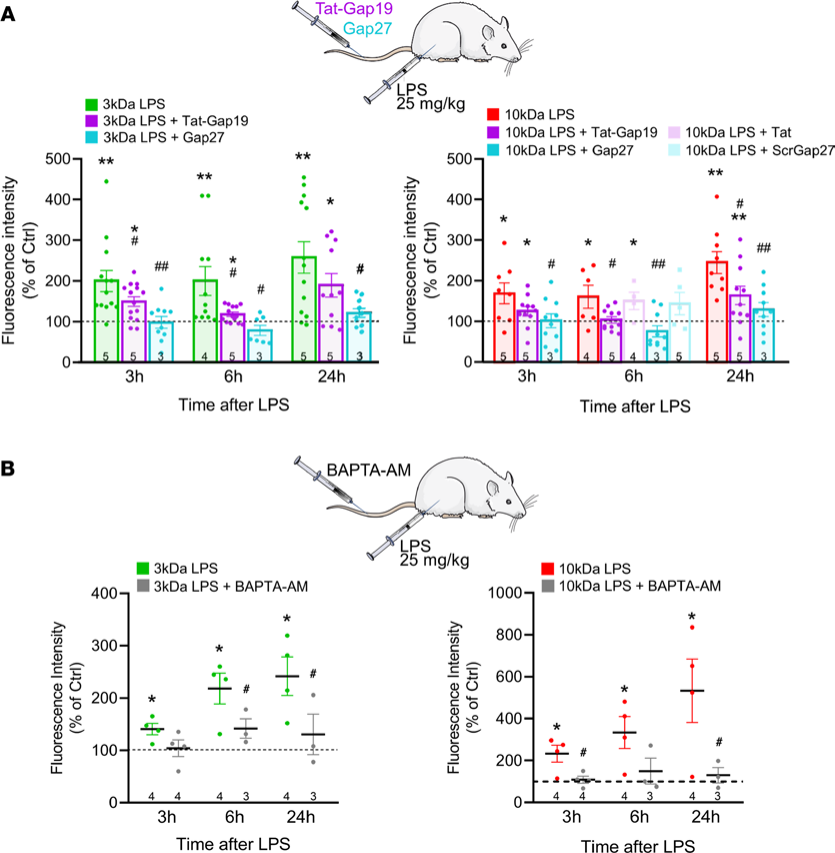
Gap27, Tat-Gap19, and intracellular Ca^2+^ chelation with BAPTA-AM reduce LPS-induced BBB leakage. (**A**) IV injection of Gap27 (25 mg/kg) or Tat-Gap19 (54 mg/kg) immediately (<1 minute) before IP administration of LPS (25 mg/kg) prevented BBB leakage to 3 kDa DF and 10 kDa DTR. Scrambled Gap27 or Tat peptide not linked to Gap19 had no effect at 6 hours 10 kDa. Stars indicate significant elevation above Ctrl (1-sample *t* test); number signs indicate significant reduction compared with LPS only (1-way ANOVA, Dunnett test). (**B**) IV injection of the intracellular Ca^2+^ chelator BAPTA-AM, 30 minutes prior to LPS, reduced the 3 and 10 kDa tracer leakage. Stars indicate significant elevation above Ctrl (1-sample *t* test); number signs indicate significant reduction compared with LPS only (2-sample *t* test). *P < 0.05, **P < 0.01, ^#^*P* < 0.05, ^##^*P* < 0.01.

To investigate the role of Cx43 in BBB leakage in vivo, Gap27 and Tat-Gap19 peptides were administered IV immediately (<1 minute) before challenging the animals with IP injected LPS. Gap27 targets an extracellular domain in the Cx43 protein sequence and acts from the outside, inhibiting hemichannels within minutes and gap junctions within hours (20–23). Tat-Gap19 binds to the intracellular C-terminal tail of Cx43 and inhibits hemichannels within minutes while not inhibiting gap junctions (reviewed in ref. 24). It crosses the BBB and reaches the astrocytes, where it associates with Cx43 (25, 26). We found that a single IV injection of Gap27 or Tat-Gap19 potently inhibited LPS-induced BBB leakage over the entire 24-hour observation window. Control experiments with scrambled Gap27 and Tat peptide not fused to Gap19 showed no effect on LPS-induced BBB leakage (Figure 3A). IV Gap27 had no significant effects on plasma IL-1β, TNF-α, IFN-γ, and IL-6 and brain IL-6; Tat-Gap19 suppressed the 3-hour plasma TNF-α increase and enhanced the 6-hour brain IL-6 elevation (Supplemental Figure 2). As the Tat-Gap19 effects were not consistent (suppressing TNF-α, enhancing IL-6), peptide inhibition of barrier leakage is unlikely to result from altered inflammatory processes at the blood or brain side.

Connexin channels are intimately linked to [Ca^2+^]_i_ signaling in BBB endothelial cells (22, 27), and we further determined the role of Ca^2+^ in LPS-induced barrier leakage using in vivo intracellular Ca^2+^ chelation with cell-permeant BAPTA-AM. Such an approach has been shown to reduce infarct volume in mouse focal cerebral ischemia (28) and to improve spatial learning in aged rats (29). Control experiments demonstrated that IV BAPTA-AM (12 mg/kg, 30 minutes before LPS) did not affect animal survival that remained at 100% for BAPTA-AM and for the DMSO vehicle (*n* = 8, data not shown). In terms of barrier function, IV BAPTA-AM treatment potently inhibited LPS-induced leakage of 3 kDa DF and 10 kDa DTR (Figure 3B). BAPTA-AM did not affect IL-1β, TNF-α, and IFN-γ but delayed the IL-6 elevation in the brain (compare Supplemental Figure 3 and Figure 1, D and E).

To determine whether prevention of BBB leakage by the peptide/BAPTA-AM treatments applied involved alterations in the tight junction proteins, we verified the effect of these experimental conditions on the expression of occludin and claudin in primary BCECs. Neither LPS alone, nor its combination with Gap27 or Tat-Gap19 treatments, had any effect on the expression levels of occludin or claudin (Supplemental Figure 4). BAPTA-AM showed a tendency to decrease claudin expression; however, this compound efficiently protected against LPS-triggered BBB leakage, bringing us to the conclusion that the observed claudin changes do not play any role in the protective effect of BAPTA-AM.

### LPS-induced barrier leakage is strongly reduced by inducible global Cx43 knockdown and Cx43/Cx30 double knockout in astrocytes.

**Figure 4:**
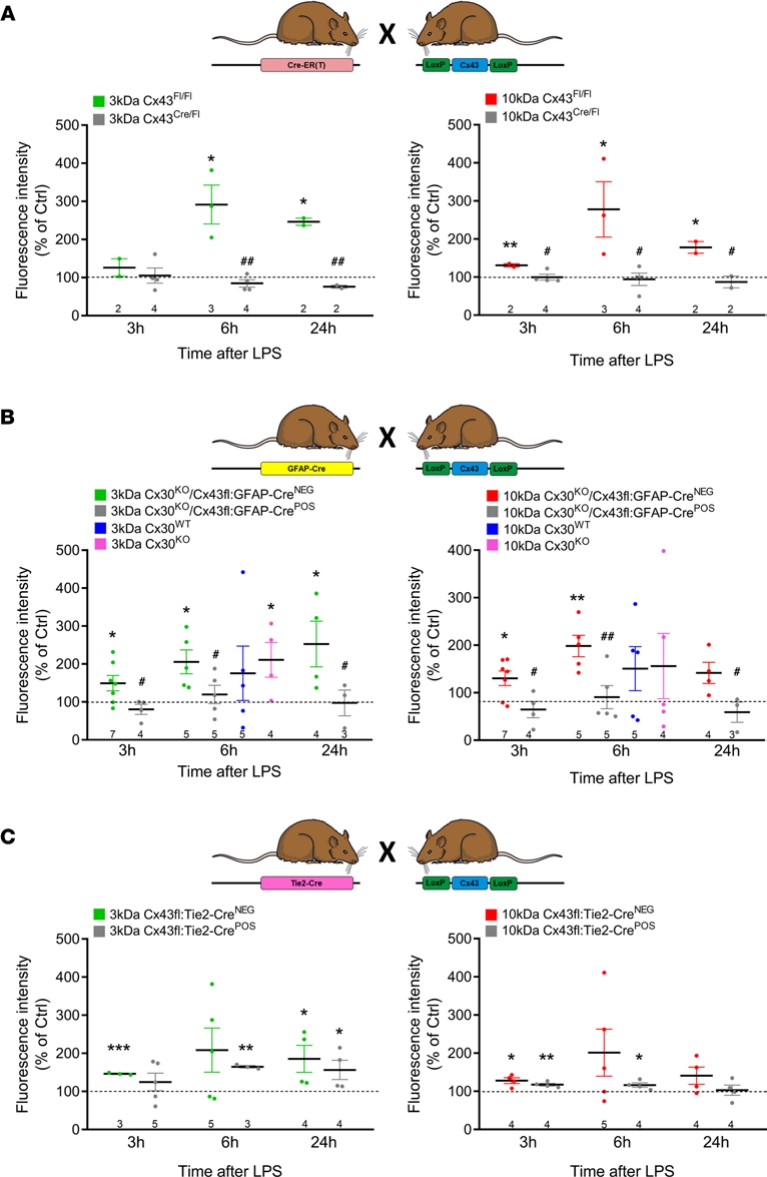
LPS-induced BBB leakage is reduced in various Cx43-knockout mice. (**A**) Effect of global Cx43 knockdown. BBB leakage in tamoxifen-treated Cx43^Cre-ER(T)/fl^ mice was strongly reduced compared with tamoxifen-treated Cx43^fl/fl^ littermates that still fully express Cx43. (**B**) Effect of astrocyte-specific Cx43 knockout combined with global Cx30 knockout (double KO) and of Cx30 knockout only. BBB leakage in Cx30^KO^/Cx43fl GFAP-Cre^+^ mice was strongly reduced compared with Cx30^KO^/Cx43fl GFAP-Cre^–^ littermates. (**C**) Effect of endothelial Cx43 knockout. Leakage in Cx43fl Tie2-Cre^+^ animals was somewhat lower compared with Cx43fl Tie2-Cre^–^ controls, but the signal did not attain statistical significance. Stars indicate significant elevation above Ctrl (1-sample *t* test); number signs indicate significant reduction compared with corresponding controls (Fl/Fl in **A**; Cre^NEG^ in **B** and **C**; 2-sample *t* test). *P < 0.05, **P < 0.01, ***P < 0.001, ^#^*P* < 0.05, ^##^*P* < 0.01.

We used different Cx43-knockout mouse lines to further substantiate the importance of Cx43 in LPS-induced barrier leakage. Tamoxifen-induced global Cx43 knockdown in Cx43^Cre-ER(T)^/Cx43^fl^ mice, exhibiting a 65% ± 5.0% (*n* = 3; measured in heart) reduction in Cx43 after tamoxifen treatment, were entirely protected against LPS-induced barrier leakage for 3 and 10 kDa tracers at all time points (Figure 4A).

For astrocytes, we used mice with conditional astrocytic Cx43 deletion under control of the glial fibrillary acidic protein (GFAP) promotor (GFAP-Cre/Cx43^fl/fl^) combined with global Cx30 knockout to prevent compensatory astrocytic Cx30 upregulation, also referred to as astrocytic Cx43/Cx30 double-knockout mice (30, 31). These double-knockout animals, displaying a 48% ± 7% (*n* = 3) reduction of astrocytic Cx43 expression (Supplemental Figure 5A), were protected against LPS-induced barrier leakage (Figure 4B). By contrast, Cx30-knockout mice still displayed leaky barriers 6 hours after LPS administration, similar to WT mice (Figure 4B). Baseline BBB function was normal in these animals (3 kDa tracer leakage in WT: 20,347 ± 9265 arbitrary units [a.u.]; Cx30^KO^: 22,337 ± 9109 a.u.; Cx30^KO^ GFAP-Cre^–^: 29,629 ± 7331 a.u.; Cx30^KO^ GFAP-Cre^+^ littermates: 31,692 ± 5681 a.u.; *n* = 3–5), as reported by others (6, 32); under high-pressure conditions leakage may occur, however (6, 33).

Conditional endothelial Cx43-knockout mice were obtained by crossing mice carrying *loxP* sites flanking exon 2 of the Cx43 gene (Cx43^fl/fl^ mice) with mice expressing Cre under control of the endothelial Tie2 receptor tyrosine kinase gene Tie2 promoter. Western blotting studies demonstrated a 64% ± 4% (*n* = 5) reduction of Cx43 expression in BCECs isolated from Cx43fl Tie2-Cre^+^ mice relative to Cx43fl Tie2-Cre^–^ control mice (Supplemental Figure 5B). LPS-induced barrier leakage tended to be decreased in Cx43fl Tie2-Cre^+^ mice (Figure 4C), but the effect did not attain statistical significance when compared to Cx43fl Tie2-Cre^–^ animals. Possibly, this may relate to an observed upregulation of Cx37 expression in primary BCECs from Cx43fl Tie2-Cre^+^ animals (196% ± 83.9%; *n* = 3; *P* = 0.024).

### LPS triggers rapid endothelial hemichannel opening that is suppressed by Gap27, Gap19, and intracellular Ca^2+^ chelation.

The fact that a single IV injection of the rapidly cleared hemichannel-inhibiting Tat-Gap19 peptide (~17.5 minutes half-life in the blood, calculated as described in Mathur et al., 2018, ref. 34) prevented barrier leakage up to 24 hours later indicates that early hemichannel opening may be crucial in the event cascade. We thus determined whether LPS could directly stimulate Cx43 hemichannel opening in addition to its activation by TNF-α and IL-1β that has been characterized previously (35, 36). We made use of patch-clamp experiments on HeLa cells overexpressing Cx43 (HeLaCx43) and on RBE4 cells derived from rat brain endothelium. In HeLaCx43 cells, voltage steps to +70 mV activated unitary current activity characterized by a unitary conductance (γ) of about 220 pS, corresponding to hemichannel opening (Supplemental Figure 6A). Within 120 seconds after LPS (1 μg/mL, applied via a fast local perfusion system), unitary current activity significantly increased, resulting in a doubling of membrane charge transfer (Q_m_) compared with baseline (Supplemental Figure 6B). Quantification of tail current hemichannel closing events upon repolarization (see dashed boxes in Supplemental Figure 6A) supported a rapid increase in hemichannel activity with LPS (Supplemental Figure 7A). LPS-enhanced hemichannel activity rapidly reverted to baseline within 40 seconds after washout (Supplemental Figure 6B). Control experiments without LPS challenging or with LPS challenging of WT HeLa cells not overexpressing Cx43 had no effect (Supplemental Figure 6D and Supplemental Figure 7, B–D). Gap19 (applied via the whole-cell patch pipette and therefore without Tat internalization sequence) and Gap27 (bath solution) strongly inhibited the LPS-enhanced hemichannel opening (Supplemental Figure 6, C–F; and Supplemental Figure 7, D, F, and G). LPS also rapidly triggered [Ca^2+^]_i_ transients in HeLaCx43 cells (Supplemental Figure 7E), and to test whether [Ca^2+^]_i_ signaling played a role stimulating hemichannel opening, we loaded the cells with BAPTA (10 mM, added via the patch pipette) and found it to strongly inhibit LPS-enhanced hemichannel activity (Supplemental Figure 6, C and D, and Supplemental Figure 7D).

**Figure 5:**
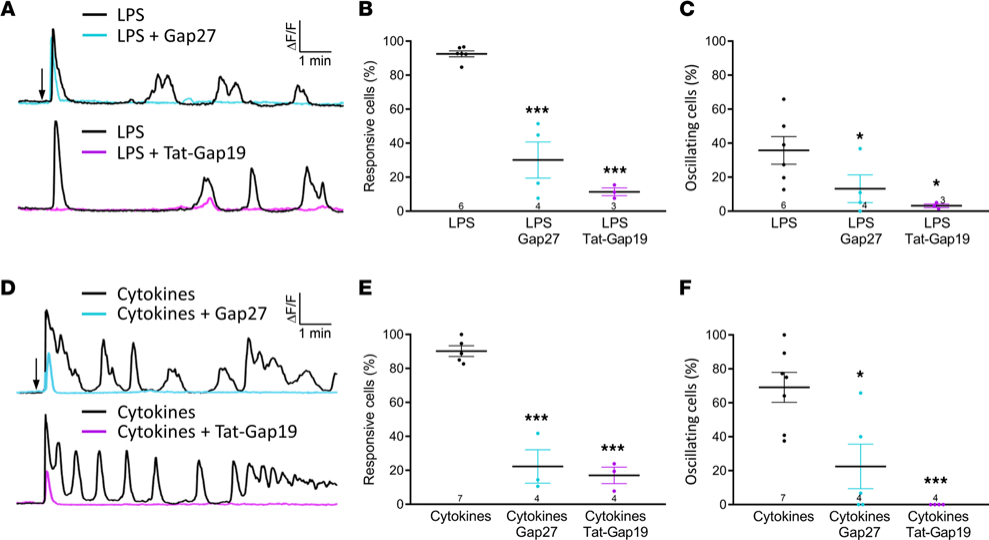
LPS and proinflammatory cytokines elicit [Ca^2+^]_i_ dynamics in RBE4 cells. (**A** and **D**) Example traces of [Ca^2+^]_i_ dynamics triggered by 1 μg/mL LPS (**A**) or a mix of proinflammatory cytokines (**D**) and the effect of Gap27 or Tat-Gap19 (200 μM, 30 minutes preincubation and present during recording). Arrow marks addition of LPS/cytokines (TNF-α, IL-1β: 200 pg/mL, IL-6: 15 ng/mL, IFN-γ: 5 ng/mL as measured in LPS-treated mice). (**B**, **C**, **E**, and **F**) Percentages of LPS/cytokine-responsive cells (**B** and **E**) and oscillating cells (**C** and **F**) were significantly reduced by Gap27 and Tat-Gap19. Stars compare with LPS/cytokine mix (1-way ANOVA, Dunnett test). *P < 0.05, ***P < 0.001.

**Figure 6:**
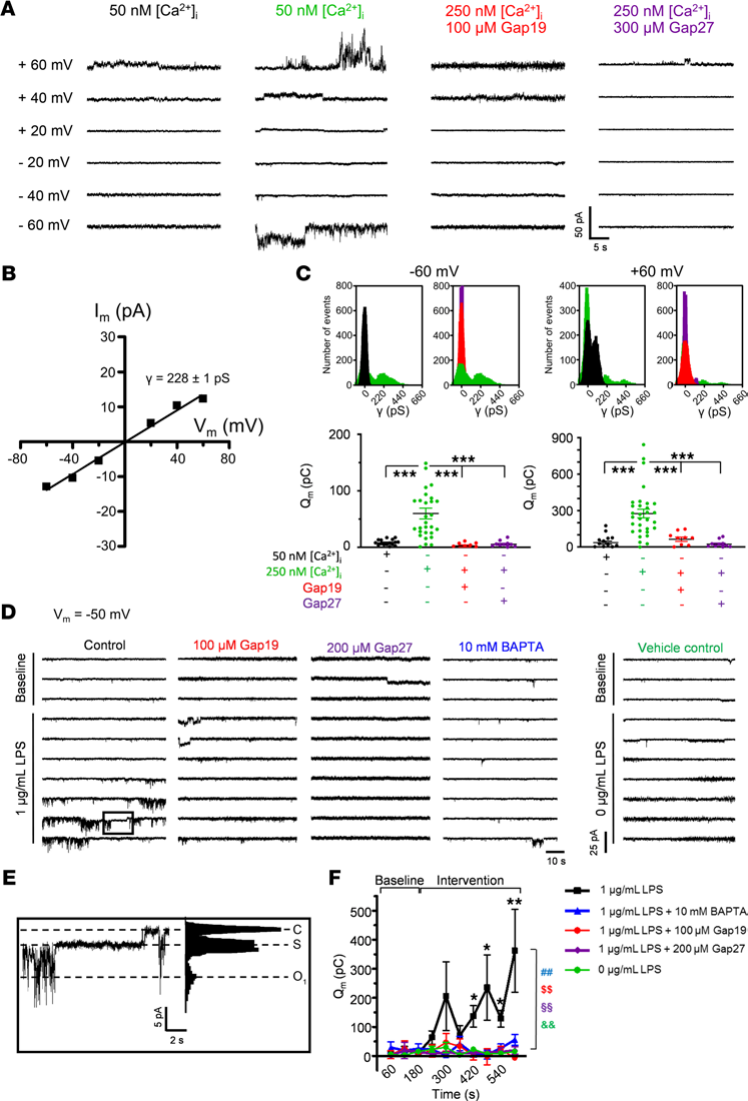
Voltage-, [Ca^2+^]_i_-, and LPS-dependent activation of Cx43 hemichannels in RBE4 cells. (**A**) Example traces depicting unitary current activities during 30-second voltage steps under conditions of 50 and 250 nM [Ca^2+^]_i_. At 250 nM, activity was present at both positive and negative voltages, which was inhibited by Gap19 and Gap27. (**B**) I-V plot for 250 nM unitary currents, demonstrating a slope conductance of 228 ± 1 pS (*n*_cells_ = 48, 5 independent experiments), characteristic for Cx43 hemichannels. (**C**) All-point histograms for unitary activities and Q_m_ summary data at –60 and +60 mV (color codes as in **A**; *n*_cells_ = 10–31 per condition, 5 independent experiments), showing [Ca^2+^]_i_-dependent current activation that is blocked by Gap19 and Gap27 (1-way ANOVA, Bonferroni test). (**D** and **E**) Consecutive current traces obtained at –50 mV (60 seconds) with and without 1 μg/mL LPS and corresponding all-point histogram (**E**). LPS induced periodic burst opening of about 210 pS unitary current activity (O_1_ in histogram), including a longer lasting substate of about 60 pS (S in histogram). The activity was blocked by Gap19 or 10 mM BAPTA in the patch pipette or bath-applied Gap27 (30 minutes’ preincubation). C, closed state. (**F**) Q_m_ summary data of **D** (*n*_cells_ = 8 per condition; 4 independent experiments). Stars compare LPS time points versus baseline at 60 seconds (repeated measures ANOVA, Dunnett test). Colored symbols compare LPS with control without LPS (green) and interventions with Gap27 (purple), Gap19 (red), or BAPTA (blue) at the last 600-second time point (1-way ANOVA, Dunnett test). *P < 0.05, **P < 0.01, ***P < 0.001, ^##^*P* < 0.01, ^$$^*P* < 0.01, ^§§^*P* < 0.01, ^&&^*P* < 0.01.

We next verified LPS effects in RBE4 cells, aiming to determine whether this could activate Cx43 hemichannel opening at normal negative resting potential. As observed in HeLaCx43 cells, LPS rapidly triggered [Ca^2+^]_i_ changes that were oscillatory in about one-third of the cells (Figure 5, A–C). These [Ca^2+^]_i_ responses were inhibited by Gap27 and Tat-Gap19, indicating they are related to hemichannel opening (Figure 5, B and C). Addition of a mix of cytokines (TNF-α, IL-1β, IL-6, IFN-γ) that were elevated in the blood after LPS (Figure 1D) also triggered [Ca^2+^]_i_ responses that were oscillatory in about two-thirds of the cells (Figure 5, D–F). We subsequently tested whether hemichannel activity could be provoked by electrical stimulation over the –60 to +60 mV range and verified the effect of 50 and 250 nM [Ca^2+^]_i_ (imposed through the patch pipette). In the 50 nM [Ca^2+^]_i_ control condition, unitary current activities only appeared at +60 mV; at 250 nM [Ca^2+^]_i_, unitary current activity also appeared at negative membrane potential while currents at positive potential were enhanced (Figure 6A). A current-voltage (I-V) plot of current amplitudes indicated a slope conductance of 228 ± 1 pS, typical for Cx43 hemichannels (Figure 6B). Gap19 and Gap27 potently inhibited unitary currents at both negative and positive potentials (Figure 6A). All-point histograms of unitary current activities at –60 mV and +60 mV (Figure 6C) indicated a single-channel conductance in the range of the slope conductance obtained from the I-V plot. Figure 6C summarizes Q_m_ data demonstrating 250 nM Ca^2+^ stimulation of hemichannel activity as well as Gap27/Gap19 inhibition for negative and positive potentials. We further tested whether LPS could directly trigger hemichannel opening under low [Ca^2+^]_i_ buffering conditions (0.1 mM EGTA in patch pipette instead of 2 mM) and with the cells held at –50 mV. Most interestingly, we found that LPS (1 μg/mL) triggered current activity with an approximately 210 pS unitary conductance and approximately 60 pS substate characteristic of Cx43 hemichannels (Figure 6, D and E); extracellular solution without LPS had no effect (Figure 6D, vehicle control). Gap19, Gap27, and intracellular BAPTA (10 mM in the pipette) all abolished the LPS-induced hemichannel currents (Figure 6, D and F).

### LPS induces astrogliosis that is prevented by Tat-Gap19 and BAPTA-AM.

**Figure 7:**
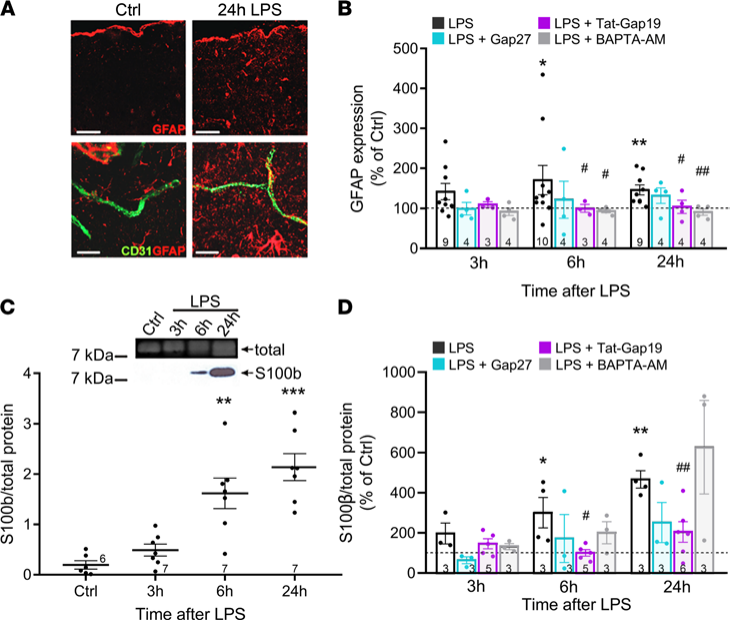
LPS triggers astrogliosis that is inhibited by Tat-Gap19 and BAPTA-AM. (**A**) GFAP immunostaining in the somatosensory cortex demonstrating LPS-induced astrogliosis. Scale bars: 100 µm (upper panels), and 10 µm (lower panels). (**B**) IV injection of Tat-Gap19 or BAPTA-AM prior to LPS reduced astrogliosis at 6 hours and 24 hours post-LPS while Gap27 had no effect. Stars indicate significant elevation above Ctrl (1-sample *t* test); number signs indicate significant reduction compared with LPS only (1-way ANOVA, Dunnett test). *P < 0.05, **P < 0.01, ^#^*P* < 0.05, ^##^*P* < 0.01. (**C**) The glial inflammation marker S100β appeared in the plasma in response to LPS and was significantly elevated from 6 hours on (1-way ANOVA, Dunnett test). **P < 0.01, ***P < 0.001. (**D**) Circulating S100β was reduced by Tat-Gap19 but not by Gap27 or BAPTA-AM (stars, number signs, and comparisons as defined in **B**).

IP administered LPS and the associated systemic cytokine surge will first activate the BBB endothelium, but astrocytes will subsequently become involved and may, as reactive astrocytes, further impair barrier function. Reactive astrocytes display increased GFAP gene expression (37), and immunohistochemical analysis supported significantly elevated GFAP signal starting from 6 hours post-LPS and persisting up to 24 hours (Figure 7, A and B). Treatment with IV administered BAPTA-AM and Tat-Gap19 prevented the GFAP increase while the less permeable Gap27 had no effect (Figure 7B). Tat-Gap19 enhanced the 6-hour brain IL-6 elevation (Supplemental Figure 2B), and prevention of GFAP elevation by Tat-Gap19 therefore does not seem to link to IL-6. S100β, an 11 kDa soluble Ca^2+^-binding protein, is another astrogliosis marker that is released from astrocytes in inflammation-linked brain disorders (38–40), as well as by LPS (41), proinflammatory cytokines (42, 43), and elevated [Ca^2+^]_i_ (44). We found significantly increased S100β levels in the plasma starting at 6 hours post-LPS and increasing to the 24-hour time point (Figure 7, C and D). As observed for GFAP, S100β appearance in the plasma was inhibited by Tat-Gap19 but not by Gap27; in contrast to GFAP, IV BAPTA-AM had no effect (Figure 7D).

### BAPTA-AM and Tat-Gap19 applied directly to the cortical surface via a cranial window prevent barrier leakage.

**Figure 8:**
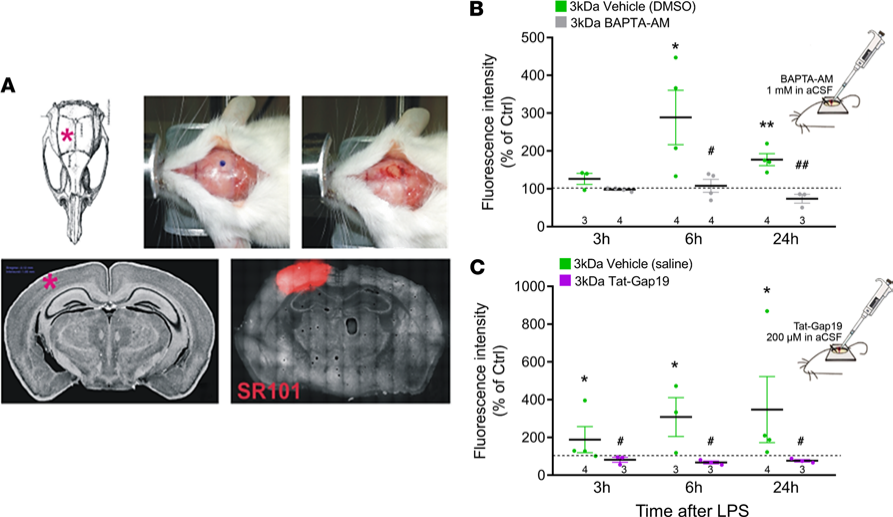
Application of BAPTA-AM or Tat-Gap19 via a cranial window prevents LPS-induced BBB leakage. (**A**) A craniotomy was made in the right parietal bone to create a window (3 mm diameter), centered 2 mm posterior to the bregma and 2 mm from the sagittal suture/midline (red star in skull and lower left image). The loaded zone was visualized by applying sulforhodamine 101 (SR101) (50 μM) to the exposed cortex, and barrier leakage was assessed in this zone only (red zone in lower right image). (**B** and **C**) Application of BAPTA-AM or Tat-Gap19 to the exposed cerebral cortex (30 minutes prior to IP LPS injection) significantly suppressed leakage of 3 kDa DF. Stars compare LPS effect with Ctrl (1-sample *t* test); number signs compare intervention (BAPTA-AM, Tat-Gap19) with LPS (vehicle applied to cortex; 2-sample *t* test). *P < 0.05, **P < 0.01, ^#^*P* < 0.05, ^##^*P* < 0.01.

To further identify the role of brain parenchymal [Ca^2+^]_i_ changes and astrocytic Cx43 hemichannels in BBB leakage, we performed experiments with BAPTA-AM and Tat-Gap19 directly applied to the exposed cerebral cortex (Figure 8A). The fluorescent indicator SR101 applied via a cranial window has been demonstrated to be taken up specifically by astrocytes (45, 46) and AM ester-based Ca^2+^ indicator loading via this way is an established procedure for monitoring astrocytic [Ca^2+^]_i_ dynamics (47–49). Here, we applied BAPTA-AM via the exposed cortex to determine whether astrocytic Ca^2+^ chelation could mitigate barrier leakage induced by IP administered LPS. Cortical barrier leakage was quantified in the SR101-positive zone (Figure 8A). BAPTA-AM significantly prevented LPS-induced BBB leakage of 3 kDa DF (Figure 8B), while it had no effect in non–LPS-treated control animals (BAPTA-AM: 32,178 ± 6554 a.u.; vehicle: 21,020 ± 3998 a.u.; *n* = 3, nonsignificant). Cranial window application of Tat-Gap19 also strongly protected against LPS-induced barrier leakage (Figure 8C), while it had no effect in non–LPS-treated control animals (Tat-Gap19: 33,242 ± 6443 a.u.; vehicle: 27,670 ± 1918 a.u.; *n* = 3).

### LPS triggers astroglial Cx43 hemichannel opening mediated by IL-6.

**Figure 9:**
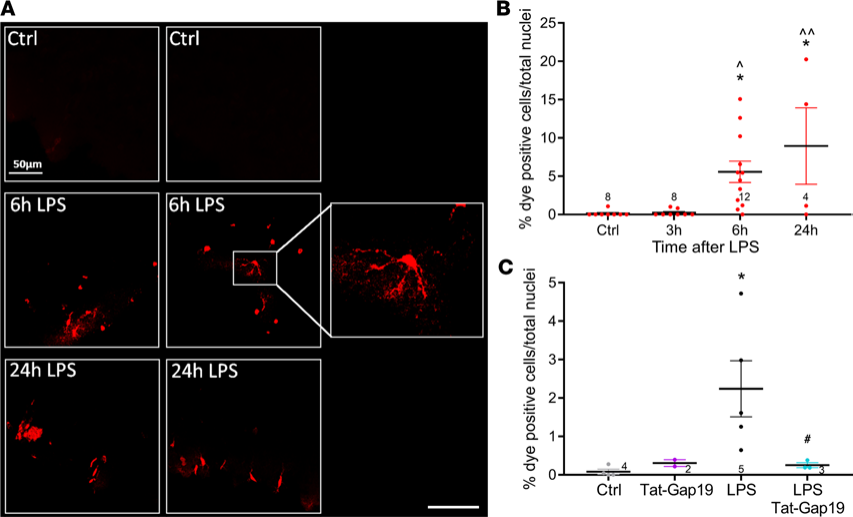
LPS triggers EtBr dye uptake that is inhibited by cortically applied Tat-Gap19. (**A**) Images illustrating cellular EtBr dye uptake after its application to the exposed cortex (100 μM, added 30 minutes before sacrifice). Scale bar: 20 μm. (**B**) The percentage of EtBr dye–positive cells increased with time after IV LPS. *P < 0.05 compared with Ctrl (1-way ANOVA, Dunnett test). Carot symbols indicate significance compared to the 3-hour time point; ^*P* < 0.05, ^^*P* < 0.01 (1-way ANOVA, Dunnett test). (**C**) Effect of Tat-Gap19 (200 μM) at the 6-hour post-LPS time point (cortically applied 30 minutes before EtBr and present with EtBr for the next 30 minutes). *P < 0.05 compared with Ctrl; ^#^*P* < 0.05 compared with LPS without Tat-Gap19 (1-way ANOVA, Bonferroni test).

To assess cortical Cx43 hemichannel opening in response to IP administered LPS, we made use of dye uptake studies whereby hemichannel-permeable ethidium bromide (EtBr) was applied in vivo on the exposed cortex followed by subsequent counting of EtBr-positive cells in isolated cortical cryosections. We observed a sharp increase in the number of dye-positive cells at 6 and 24 hours after LPS injection (Figure 9, A and B). The number of dye-positive cells was significantly reduced to baseline when Tat-Gap19 was included in the EtBr solution applied to the exposed cortex (30 minutes 200 μM Tat-Gap19 that was continued in the presence of 100 μM EtBr for the next 30 minutes) (Figure 9C).

**Figure 10:**
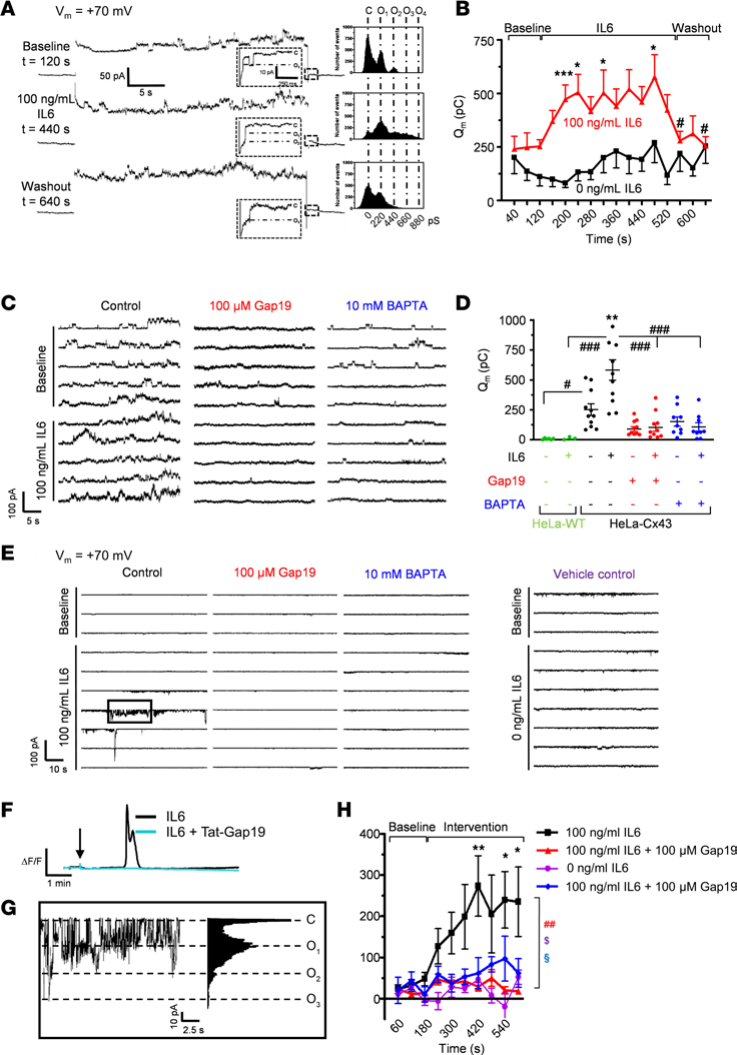
IL-6 enhances Cx43 hemichannel opening in HeLaCx43 cells and activates Cx43 hemichannels in primary cultured astrocytes in a Ca^2+^-dependent manner. (**A**) Example traces and matching all-point histograms depicting representative voltage-induced (+70 mV, 30 seconds) unitary current activity recorded in HeLaCx43 cells before (baseline), during, and after application of IL-6 (100 ng/mL) via a fast local perfusion system. Insets show hemichannel closing events in the tail currents. (**B**) Q_m_ summary data for repeated current measurements for IL-6 (100 ng/mL, red trace) and Ctrl (0 μg/mL LPS, black trace) (*n*_cells_ = 9 per concentration; 5 independent experiments). Stars compare with 40-second point (repeated measures ANOVA, Dunnett test); number signs compare with the 520-second point (repeated measures ANOVA, Dunnett test). *P < 0.05, ***P < 0.001, ^#^*P* < 0.05. Average Q_m_ during IL-6 (160–520 seconds) was significantly above Ctrl without IL-6 (*P* < 0.001; 2-sample *t* test). (**C**) Representative current traces illustrating the effect of Gap19 and BAPTA applied via the patch pipette. (**D**) Q_m_ summary data of **C** (*n*_cells_ = 9–11 per condition; 6 independent experiments). Stars compare IL-6 versus Ctrl without IL-6; number signs compare between IL-6 and conditions indicated by the lines (1-way ANOVA, Bonferroni test). **P < 0.01, ^#^*P* < 0.05, ^###^*P* < 0.001. (**E**) Consecutive current traces obtained at –70 mV (60 seconds) with and without 100 ng/mL IL-6 in primary mouse astrocytes. IL-6–induced burst unitary current activity that was inhibited by Gap19 or BAPTA added to the patch pipette. (**F**) Example traces demonstrating a typical Ca^2+^ response to IL-6 (100 ng/mL) in primary astrocytes, which was inhibited by Tat-Gap19 (200 μM, 30 minutes pretreatment). (**G**) All-point histogram of current activity in the boxed area of **E**, demonstrating unitary activity of 220 pS (O_1_) and multiples thereof (O_2_, O_3_). (**H**) Q_m_ summary data of **E** (*n*_cells_ = 10–17 per condition; 5 independent experiments). Stars compare IL-6 time points versus baseline at 120 seconds (repeated measures ANOVA, Dunnett test). Colored symbols compare IL-6 with Ctrl without IL-6 (purple) and interventions with Gap19 (red) or BAPTA (blue) at the last 600-second time point (1-way ANOVA, Dunnett test). *P < 0.05, **P < 0.01, ^##^*P* < 0.01, ^$^*P* < 0.05, ^§^*P* < 0.05.

LPS is known to indirectly trigger astrocytic hemichannel opening via activation of microglial cells that release TNF-α and IL-1β (35, 50, 51). However, systemically administered LPS is not likely to enter the brain (Banks and Robinson, 2010, ref. 52), nor did we find elevated brain TNF-α and IL-1β levels in response to systemic LPS (Figure 1E). More interestingly, we found increased brain IL-6 levels (Figure 1E), the effect of which on Cx43 hemichannels is currently unknown. We thus explored IL-6 effects on hemichannel currents in HeLaCx43 cells and primary cultured cortical astrocytes. Hemichannel currents in HeLaCx43 cells were significantly enhanced by 100 ng/mL IL-6 within 80 seconds (~2-fold increase in Q_m_ and hemichannel closing event counts) and reverted to baseline within 40 seconds after washout (Figure 10, A and B, and Supplemental Figure 8). Gap19 and BAPTA strongly inhibited these responses (Figure 10, C and D, and Supplemental Figure 8D). Cx43 hemichannel opening can be directly activated by [Ca^2+^]_i_ in astrocytes (26, 53), and we here found IL-6–induced [Ca^2+^]_i_ changes to be inhibited by Tat-Gap19, indicating a linkage to hemichannel opening (Figure 10F). We thus performed patch-clamp experiments on primary cultured astrocytes held at –70 mV under low intracellular Ca^2+^ buffering conditions (0.1 mM EGTA in patch pipette) and exposed them to IL-6 (100 ng/mL), which induced unitary current activities with a conductance as expected for Cx43 hemichannels (Figure 10, E and G). Gap19 and BAPTA significantly inhibited these responses, demonstrating Ca^2+^-dependent hemichannel activation (Figure 10, E and H).

## Discussion

**Figure 11:**
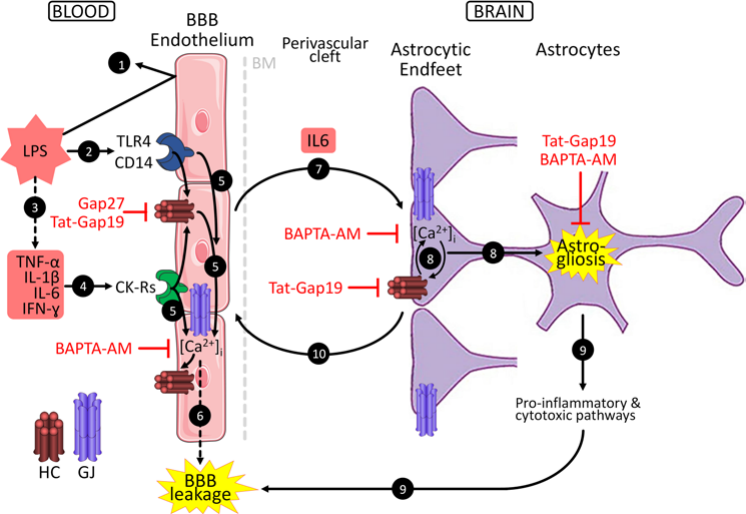
Schematic drawing summarizing the findings of this study. Numbered steps are explained in the Discussion. The perivascular cleft has been stretched for clarity. BM, basement membrane.

LPS is a frequently used experimental tool to initiate a systemic inflammatory response that spreads out to the cerebral parenchyma. LPS itself poorly penetrates into the brain (52, 54) (Figure 11 step 1) but exerts its cerebral effects via an innate immune response through activation of endothelial Toll-like receptor 4 and CD14 (12–14) (Figure 11 step 2). Additionally, LPS triggers a cytokine storm with IL-1β, TNF-α, IL-6, and IFN-γ as major proponents (55, 56) (Figure 11 step 3) that disturb BBB function (57–59) (Figure 11 step 4). We here provide evidence that LPS and circulating cytokines increase the low baseline Cx43 expression in the barrier endothelium and its high baseline expression in astrocytic endfeet. LPS/cytokines furthermore activate [Ca^2+^]_i_ dynamics and hemichannel opening (Figure 11 step 5). Cx43 hemichannels have been demonstrated to sustain [Ca^2+^]_i_ oscillations and thereby contribute to barrier leakage (22, 60), so barrier leakage likely results from endothelial LPS/cytokine effects combined with hemichannel opening (Figure 11 step 6). In vivo interfering with connexins and [Ca^2+^]_i_ by IV administration of Gap27, Tat-Gap19, or BAPTA-AM (marked red in Figure 11) prevents the LPS-induced barrier failure.

In addition to their barrier-forming role, BBB endothelial cells are a relay station in immune-brain communication by secreting immune factors and proinflammatory cytokines, in particular IL-6 as reported here. Possibly, circulating IL-6 (MW 21 kDa) may have leaked through the barrier, as suggested by the synchronized IL-6 peaks at 3 and 6 hours in plasma and brain (Figure 1, D and E). However, one would then expect leakage of other cytokines, with MW in the order of the 3 and 10 kDa fluorescent leakage markers used (IL-1β 1 kDa, IFN-γ ~17 kDa), to also appear in the brain, which was not the case. The fact that IL-6 was the only cytokine (out of 4 tested) elevated in the brain indicates that more specific mechanisms are involved. IL-6 exerts its effects via a receptor complex that consists of the IL-6 receptor (IL-6R) and the signal transduction receptor subunit gp130 (61, 62). Both gp130 and IL-6R are widely distributed throughout the brain and are upregulated by exposure to proinflammatory conditions (63, 64). IL-6 receptors are present in astrocytic endfeet (65), and their activation may induce reactive astrocytosis (astrogliosis) (66, 67). We here demonstrate that IL-6 triggers astrocytic [Ca^2+^]_i_ dynamics as well as Cx43 hemichannel opening (Figure 11 step 7). [Ca^2+^]_i_ changes interact with hemichannels in a bimodal manner (68), sustaining astrocytic [Ca^2+^]_i_ dynamics (22, 60) and thereby leading to astrogliosis (Figure 11 step 8). Reactive astrocytes on their turn upregulate proinflammatory and cytotoxic pathways, and consequently, produce a range of substances associated with barrier leakage (61, 69, 70; reviewed in ref. 67) (Figure 11 step 9). In line with this event sequence, IV administration of barrier-permeable BAPTA-AM and Tat-Gap19, prevent GFAP upregulation, which is supported by in vitro evidence for a role of astrocytic [Ca^2+^]_i_ therein (71–73).

Common vasodilatory agents that are released from astrocytic endfeet in a Ca^2+^-dependent manner during neurovascular coupling, e.g., adenosine and prostaglandin E_2_ (74, 75; reviewed in ref. 76), have known BBB-disintegrating effects and may contribute to barrier leakage (77–82) (Figure 11 step 10). Last, cortically applied Tat-Gap19 and BAPTA-AM aimed at targeting astrocytes potently inhibited barrier leakage, supporting a forefront role of astrocytic Cx43 hemichannels and [Ca^2+^]_i_ signaling in barrier leakage in response to peripheral inflammation.

IV BAPTA-AM strongly protected against BBB leakage, most likely by its first-line actions in the vascular compartment and its diffusion to subsequent cell layers, including astrocytes. Neurons also take up BAPTA-AM (28, 83), but ester-loading is in general less efficient than in astrocytes (84). The fact that IV BAPTA-AM potently prevented GFAP upregulation in astrocytes (Figure 8B) indicates excellent astrocytic BAPTA loading and tempering of [Ca^2+^]_i_ dynamics, thereby contributing to barrier protection as demonstrated by the cranial window BAPTA-AM applications that showed equally strong protection as observed with IV injection. Tat-Gap19 has an intracellular target, the C-terminal tail of Cx43 (85), and is BBB permeable. One hour after systemic administration, it is detected in GFAP-positive astrocytes where it colocalizes with Cx43 and is still detectable in the parenchyma after 24 hours (25, 26). Moreover, systemically administered Tat-Gap19 has been shown to decrease seizure activity in animal epilepsy models (86) and to prevent neuronal cell loss in Parkinson’s disease (87), indicating it is biologically active in the brain. In contrast, Gap27 has an extracellular target (extracellular loops of Cx43 and Cx37) and has no known intrinsic membrane permeability, making its entry into the barrier-intact brain unlikely. Given the short lifetime of Gap27 circulating in the blood (half-life of ~14 minutes), it is highly unlikely that the barrier would be sufficiently leaky to allow significant peptide entry into the brain. Thus, we anticipate that IV Gap27 prevents BBB leakage by inhibiting connexin channels in the luminal endothelial membranes, whereas IV Tat-Gap19 will target Cx43 hemichannels in both endothelial cells and astrocytes. IV Gap27 had an overall stronger effect against barrier leakage compared with IV Tat-Gap19, which may result from Gap27 affecting connexins other than Cx43, e.g., endothelial Cx37 that has the same EL2-located peptide sequence as for Cx43, or potential inhibition of gap junctions, an effect not exerted by Tat-Gap19. However, gap junctional inhibition by Gap27 necessitates more than 6 hours, i.e., far beyond its approximately 40 minutes (3 × half-life) anticipated residence in the blood (20, 22, 23). Thus, the stronger effect of IV Gap27 is likely the result of its extrainhibitory effect on Cx37 hemichannels as indicated by earlier work (22). The contribution of Cx37 is further suggested from the poor protection of Cx43 Tie2-Cre^+^ mice against barrier leakage, in which endothelial Cx37 expression was found to almost double, thereby obscuring Cx43-linked protection. This kind of compensatory alterations of vascular connexins upon knockout have been reported by others previously (88–91). In contrast to this, we found Cx37 to be lowered by LPS treatment, though this did not attain statistical significance (Supplemental Figure 1). A question that remains is why Tat-Gap19 had more pronounced effects on barrier leakage when applied to the cortex compared with its systemic application (compare Figure 4A with Figure 9C). Tat-Gap19 may have better bioavailability following direct loading into astrocytes as compared with systemic injection.

IV Tat-Gap19 and IV BAPTA-AM inhibited barrier leakage as well as GFAP elevation while IV Gap27 inhibited barrier leakage without tempering GFAP (nonsignificant effects, Figure 7B), indicating that astrogliosis is not necessarily a direct consequence of barrier leakage per se in the LPS model. Rather, endothelial cells, activated by LPS or circulating cytokines, communicate with astrocytes by secreting immune factors that may induce astrogliosis (66, 67). Remarkably, neither IV BAPTA-AM nor IV Gap27 inhibited S100β appearance in the blood (nonsignificant effects, Figure 7D) while IV Tat-Gap19 did. The absence of IV Gap27 effects is in line with the GFAP observations and results from poor penetration into the brain parenchyma. It is unclear why IV BAPTA-AM had no effect, as S100β, like GFAP (71–73), is activated by astrocytic [Ca^2+^]_i_ elevation (44), and BAPTA-AM is expected to reduce S100β production as well as its passage through the BBB because of its endothelial effects preventing barrier leakage (Figure 4B). Presumably, S100β activation may involve Ca^2+^-independent effects.

To summarize, our data indicate that Cx43 in BBB endothelial cells and astrocytes plays a crucial role in barrier leakage induced by systemic inflammation that is mediated by the opening of hemichannels. In the blood, LPS as well as several proinflammatory cytokines trigger endothelial hemichannel opening while in the brain, IL-6 activates astrocytic hemichannels. Cytoplasmic Ca^2+^ dynamics in these cells is intimately involved in triggering barrier leakage as a cause but also consequence of hemichannel opening. Targeting this connexin/Ca^2+^ axis in both brain endothelial cells and astrocytes is therefore an effective approach, resulting in suppression of barrier leakage as well as astrogliosis. Extending the spectrum of hemichannel inhibitor substances to vascular connexins other than Cx43 may further improve the efficiency of preventing inflammatory barrier leakage.

## Methods

A detailed description of all procedures can be found in the Supplemental Methods.

### Animals.

Experiments were performed in male FVB mice (Janvier Labs), inducible global Cx43^Cre-ER(T)/fl^ mice (provided by R Schulz, Justus-Liebig University, Giessen, Germany), global Cx30-KO and Cx30^KO^/Cx43fl GFAP-Cre “double-KO” mice (provided by Ghent University and University of Bonn), and Cx43fl Tie2-Cre endothelial Cx43-KO mice (provided by D Krysko, Ghent University).

Adult mice (25–30 g, aged 6–12 weeks) received an IP bolus injection of LPS (freshly dissolved in filter-sterilized saline, 0.9% NaCl, prior to injection). Mice received a single IV bolus injection of Gap27 (25 mg/kg; 200 μM in blood volume) or Tat-Gap19 (54 mg/kg; 200 μM in blood volume) freshly dissolved in filter-sterilized saline, immediately before IP LPS injection. Control peptides were administered in an identical manner. Vehicle control mice received an IV injection with saline only, prior to LPS. The half-life of Gap27 and Tat-Gap19 peptides was calculated with the peptide lifetime predictor (PlifePred, http://crdd.osdd.net/raghava/plifepred/) as described (34). BAPTA-AM was prepared at a concentration of 33 mM in DMSO and 0.16% pluronic acid and further diluted in sterile saline to a final concentration of 2 mM or 12 mg/kg (+0.01% pluronic acid). BAPTA-AM was administered IV, 30 minutes prior to IP LPS injection. Vehicle control mice received a bolus injection of DMSO + pluronic acid dissolved in sterile saline.

### BBB permeability.

Ten minutes prior to decapitation, mice received an IV bolus (200 μL) of 3 kDa DF (30 mg/kg), 10 kDa DTR (100 mg/kg), or FITC-albumin (66 kDa; 660 mg/kg), through tail vein injection, each dye ultimately reaching an estimated 200 μM in the blood compartment. Subsequently, animals were transcardially perfused, and brains were isolated and snap-frozen. Parenchymal fluorescence was quantified in coronal brain cryosections and expressed relative to the signal observed in nontreated control animals (number of animals as in the treated group).

### Cranial window.

A craniotomy was made in the right parietal bone covering the somatosensory cortex (for details, see Supplemental Methods), and artificial CSF containing SR101 (50 μM) to mark astrocytes was applied to the exposed cortex. For interventions targeting [Ca^2+^]_i_ or Cx43 hemichannels, BAPTA-AM (2 mM) or Tat-Gap19 (200 μM) were included in the solution. Subsequently, LPS was injected IP and animals were allowed to recover. At 3, 6, and 24 hours mice were anesthetized for barrier permeability measurements as described above. Cortical fluorescence intensity was measured in the SR101-loaded region.

For cortical hemichannel dye uptake experiments, EtBr (100 μM) was added to the exposed cortex 30 minutes before sacrifice. Thereafter, mice were transcardially perfused. EtBr-positive cells were counted in coronal brain sections and expressed relative to the number of nuclei.

### Electrophysiological recording.

See Supplemental Methods.

### Calcium imaging.

Cells were loaded with the Ca^2+^ indicator dye fluo3-AM and transferred to an inverted epifluorescence microscope (Eclipse TE 300, Nikon Belux) equipped with a superfusion system that allowed fast solution switching. Ca^2+^ oscillations were counted in a 10-minute observation period and were defined as at least 2 transient Ca^2+^ changes after the initial Ca^2+^ transient in a single cell, minimally 5% above baseline fluo3 fluorescence.

### Immunohistochemistry.

Immunohistochemical analysis of protein expression was performed on coronal mouse brain cryosections or freshly isolated capillaries (see *Cell isolation and cell culture studies* in Supplemental Methods, excluding enzymatic digestion and subsequent steps). Primary antibodies were rat anti-CD31 (Invitrogen catalog RM5200 or BD Biosciences catalog 550274), rabbit anti-AQP4 (CT epitope, Merck/Sigma-Aldrich catalog AB3594), rabbit-GFAP (polyclonal, Abcam catalog ab7260), GFAP-Cy3 (monoclonal, Invitrogen catalog C9205), rabbit anti-Cx43 (MilliporeSigma catalog C6219), and mouse anti-Cx43 (Merck Millipore/BD Biosciences catalog MAB3067). Further details on antibodies used can be found in the Supplemental Methods.

### Gel electrophoresis and Western blotting.

Cell lysates and plasma samples were separated by electrophoresis over a 4%–12% SDS-PAGE and transferred to a nitrocellulose membrane. Blots were probed with rabbit anti-Cx43 antibody (MilliporeSigma) or rabbit anti-S100β (Abcam). Quantification was done using ImageJ (NIH). Other antibodies used in Western blotting experiments presented as Supplemental Methods are specified there.

### Statistics.

In all figures, data are expressed as mean ± SEM with *n* giving the number of independent experiments (indicated on the corresponding bar), and statistical analysis was performed using GraphPad Prism software. Occasional outliers were removed making use of GraphPad QuickCalcs Outlier calculator. Normality of the distributions was verified with the Kolmogorov-Smirnov test; all data were normally distributed except for the IL-1β and TNF-α data that deviated from normality. Data expressed relative to data obtained in an associated nontreated control group (Figure 1C; Figure 2A; Figure 3; Figure 7, B and D; and Figure 8) were compared to the control 100% level by a 1-tailed *t* test. Two groups were compared with a 2-tailed *t* test. Multiple groups were compared by 1-way ANOVA with Bonferroni post hoc testing; Dunnett testing was used for repeated comparisons to a defined control condition. Time series data were compared by repeated measures ANOVA with Dunnett post hoc test. Nonparametric Kruskal-Wallis testing was done for IL-1β and TNF-α data (Figure 1D and Supplemental Figure 1A). Results were considered statistically significant when *P* < 0.05 (1 symbol for *P* < 0.05, 2 for *P* < 0.01, and 3 for *P* < 0.001).

### Study approval.

Mice were treated according to the European Ethics Committee guidelines, and the study protocol was approved by the animal experiment ethical committee of the Faculty of Medicine and Health Sciences (Ghent University).

## Author contributions

MDB, MDS, SV, HT, SS, and VVH designed and conducted experiments; KW, CS, NR, and REV provided experimental and analytical tools and expertise; MDB and LL wrote the manuscript with input from all authors; MDB and LL supervised the project. Co–first authorship was decided based on leadership (MDB) and crucial contributions to functional hemichannel assessment and experiments (MDS).

## Supplementary Material

Supplemental data

## Version 1 07/26/2022

In-Press Preview

## Version 2 08/22/2022

Electronic publication
