# Supplementary material for: Targeting gliovascular connexins prevents inflammatory blood-brain barrier leakage and astrogliosis
Source: JCI Insight. 2022 Aug 22;7(16):e135263. doi: 10.1172/jci.insight.135263 (PMC9462469; doi:10.1172/jci.insight.135263)
Supplement: Supplemental data [file jciinsight-7-135263-s156.pdf]

SUPPLEMENTAL DATA

Methods

Chemicals and reagents

Agarose, 1,2-bis-(2-aminophenoxy)-ethane-*N,N,N′,N′*-tetraacetic acid acetoxy methyl ester (BAPTA-AM), Ethidium Bromide, Fluo3-AM, 3 kDa dextran fluorescein (lysine-fixable) and 10 kDa dextran texas red (lysine-fixable), Pluronic acid, were from Molecular probes (ThermoFisher Scientific). FITC-albumin, heparin, LPS from E. coli 055:B5 (L2280), probenecid, tamoxifen were from Sigma-Aldrich. Gap27 peptide (SRPTEKTIFII) and Tat-Gap19 peptide (YGRKKRRQRRR-KQIEIKKFK) were obtained from Pepnome Ltd. (Hong Kong) at > 90 % purity.

Genotyping of conditional KO animals

DNA was extracted from tail samples (obtained at the age of 4 weeks) using the KAPA express kit (KAPA Biosystems), cDNA was amplified using the KAPA2G Fast Genotyping kit (KAPA Biosystems) according to the manufacturer's instructions. All primers were synthesized by ThermoFisher Scientific. The PCR end-products were separated on a 1.5 % agarose gel and visualized with SYBR Safe DNA gel stain (ThermoFisher Scientific).

Cx43 knockdown in inducible Cx43Cre-ER(T)/fl mice (kindly provided by Dr. R. Schulz, Justus-Liebig University, Giessen, Germany) was achieved by intraperitoneal (IP) injection of 1 mg tamoxifen during 5 consecutive days and experiments were performed on the 10th day. Cx43Cre-ER(T) negative/fl mice received the same tamoxifen treatment as their Cx43Cre-ER(T) positive littermates. Floxed Cx43 was detected using primers UMP (5' TCATGCCCGGCACAAGTGAGAC 3') and UMPR (5' TCACCCCAAGCTGACTCAACCG 3') giving a 1100 bp product. Presence of Cre-ER(T) was confirmed by primers Cre26 (5' CCTGGAAAATGCTTCTGTCCG 3') and Cre36 (5' CAGGGTGTTATAAGCAATCCC 3') giving a 400 bp amplicon.

GFAP-Cre Cx43fl/fl $Cx30^{-/-}$ mice (Lutz et al., 2009; Wallraff et al., 2006), transgenic mice with a conditional deletion of the Cx43 gene *Gja1* in astrocytes under control of the GFAP

promoter (Theis et al., 2003) crossed with mice exhibiting an unrestricted deletion of the Cx30 gene *Gjb6* (Teubner et al., 2003). The presence of GFAP-Cre was detected using primers GFAP-LZ1 (5' ACTCCTTCATAAAGCCCTCG 3') and Cre-LZ4 (5' ATCACTCGTTGCATCGACCG 3') rendering a 200bp product. For the simultaneous detection of Cx43 floxed alleles (650 bp amplicon) and Cx43 wild-type alleles (500 bp amplicon) we combined 43DelFwd primer (5' GGCATACAGACCCTTGGACTCC 3') with UMPR primer (5' TCACCCCAAGCTGACTCAACCG 3'). The Cx30 KO PCR was performed with a primer triplet including Cx30wt5 (5' GGTACCTTCTACTAATTAGCTTGG 3'), Cx30wt3 (5' AGGTGGTACCCATTGTAGAGGAAG 3') and Cx30LacZ3 (5' AGCGAGTAACAACCCGTCGGATTC 3').

Cx43fl:Tie2Cre mice were kindly provided by Dr. D. Krysko (Ghent University, Belgium). For detection of the Tie2-cre transgene, primers Tie2-Cre Fwd (5' GCCTGCATTACCGGTCGATGCAACGA 3') and Tie2-Cre Rev (5' GTGGCAGATGGCGCGGCAACACCATT 3') were used, generating an 800 bp amplicon. For simultaneous detection of the Cx43 floxed allele and the Cx43 wild-type allele, primers Cx43Fl-Fwd (5' GTCTCACTGTTACTTAACAGCTTGA 3') and Cx43Fl-Rev (CTTTGACTCTGATTACAGAGCTTAA) were applied (Cx43flox PCR), generating a 580 bp floxed amplicon and a 490 bp wild-type amplicon. For detection of the deleted Cx43 allele, primers (5' GCTACTTCTTGCTTTGACTCTGATTA 3') and (5' GCTCACTTGATAGTCCACTCTAAGC 3') were used (Cx43del PCR), generating a 686 bp amplicon.

Animal dose response curve for LPS treatment

A dose response experiment with concentrations ranging between 1 mg/kg and 50 mg/kg was performed to estimate the optimal dose for further experiments, which was 25 mg/kg (corresponding to ~300 µg/mL blood). This dose leads to clear sickness behavior including decreased motor activity, withdrawal, and reduced food and water intake (Dantzer et al., 2008). As inflammation progressed, animals displayed lack of grooming (fuzzy fur), diarrhea and bleared eyes.

Blood-brain barrier permeability

All dyes were dissolved in Hank's Balanced Salt Solution (HBSS) containing $CaCl_2$ (1.26 mM), $MgCl_2$ (0.49 mM), $MgSO_4$ (0.41 mM), NaCl (137.93 mM), Na_2HPO_4 (0.34 mM), KCl (5.33 mM), KH_2PO_4 (0.44 mM), D-glucose (5.56 mM) and Hepes (25 mM).

Mice were anesthetized with a mixture of ketamine (20 mg/mL) and xylazine (0.1 %). The animals were intracardially injected with 10 µL heparin (2000 U/mL) and transcardially perfused with PBS. Immediately after isolation, the brains were snap-frozen in liquid nitrogen-cooled isopentane and stored at -80 °C. Fifty micron thick coronal brain sections were cut using a using a cryostat (Leica Biosystems) and mounted in Vectashield antifade mounting medium containing Dapi stain (Vector Labs). Banks et al. previously indicated that the brain shows regional variability in terms of LPS-induced BBB permeability increases with cortical area's showing a 20-55 % increase (Banks et al., 2015). The area from which sections were made was located between 0.94 mm and 2.5 mm posterior to the bregma, where the cortical region corresponds to the primary somatosensory cortex. Parenchymal fluorescence in the brain sections was visualized using a BD Pathway 435 BioImaging system (Becton Dickinson) equipped with a mercury light source that provides illumination from 360 nm to 700 nm, CCD camera and 10x objective. The signal intensity was determined using ImageJ software. For each section, fluorescence intensity was determined in 20 points, all located in the somatosensorial cortex. Background fluorescence was measured just outside the coronal sections and was subtracted from the fluorescence in the measurement points. To reduce variation among different experiments, results are expressed relative to control non-treated animals included in the same experiment.

Cranial window

Ketamine/Xylazine-anesthetized mice were fixed in a stereotactic frame. Using a dental drill, a craniotomy was made in the right parietal bone covering the somatosensorial cortex. The cranial window was 3 mm in diameter, centered 2 mm posterior to the bregma and 2 mm from the sagittal suture/midline. The dura was carefully removed, and the exposed cortex was loaded with artificial cerebrospinal fluid (aCSF; in mM: 1 $CaCl_2$; 2 $MgCl_2$; 126 NaCl; 1.25 Na_2HPO_4; 2.5 KCl; 10 D-glucose and 25 Hepes) containing sulforhodamine 101 (SR101, 50 µM) further supplied with BAPTA-AM (2 mM) or Tat-Gap19 (200 µM) for interventions directed at

intracellular Ca^{2+} chelation or Cx43 hemichannel inhibition. After incubation of active compounds or vehicle, agarose (0.5% in aCSF) was used to seal the cranial window and the overlying skin was sutured. Subsequently, LPS was injected IP and animals were allowed to recover. At the indicated time points after LPS injection (3, 6 and 24 h), mice were anesthetized again for barrier permeability measurements as described above. Cortical fluorescence intensity was only measured in the SR101 loaded region.

Cortical hemichannel dye uptake experiments

LPS was injected IP and vehicle (aCSF) or Tat-Gap19 was applied to the exposed cortex 1 h prior to sacrification. After 30 min, ethidium bromide (EtBr, 100 µM) was added to the solution for the next 30 min. Next, mice were transcardially perfused with PBS, brains were collected in 4% PFA and subsequently snap-frozen in liquid nitrogen-cooled isopentane. Twenty micron thick sections were cut and mounted in Dapi-containing Vectashield. EtBr uptake was visualized using a Leica SP8 confocal microscope.

Plasma and brain tissue preparation for luminex assay

Whole blood samples, obtained by cardiac puncture (prior to perfusion with PBS) and collected in heparinized test tubes, were centrifuged at 2000 g at 4 °C and for 15 min to deplete cells and platelets. The resulting supernatant (plasma) was carefully removed from the cell pellet and stored at -20°C. Brain samples were lysed in CHAPS buffer (3 mL/g tissue) containing CHAPS (0.5%), Hepes (10 mM), KCl (42 mM) and $MgCl_2$ (5 mM). DTT (1 mM) and protease inhibitor cocktail (20 µL/mL) were freshly added prior to use. Protein concentration was determined using the Biorad DC protein assay kit (BioRad, Nazareth, Belgium) and absorbance was measured with a 590 nm long-pass filter.

Quantification of cytokines (TNFα, IL1β, IFNγ and IL6) in plasma and brain tissue lysates was performed using the BioPlex cytokine assay (BioRad), according to the instructions of the manufacturer. Circulating cytokine levels are expressed as pg/mL plasma, cerebral cytokine levels are expressed as pg/100µg total protein.

Cell isolation and cell culture studies

Isolation and culturing of primary mouse brain capillary endothelial cells was performed as described in (Coisne et al., 2005) with slight adaptations. Whole brains from decapitated C57BL/6 male mice (12 weeks) were collected in ice-cold phosphate buffered saline (PBS). Meninges and large extracerebral vessels were removed using sterile lint and dry cotton swabs. Optic nerves, white matter and cerebellum were carefully dissected away. The remaining tissue was grounded in ice-cold Hank's Balanced Salt Solution (HBSS) containing 10 mM Hepes and 0.1 % BSA ('WBB'), using a 15 mL Dounce homogenizer (Wheaton, Fisher Scientific), first with loose and then tight pestle. The resulting homogenate was mixed with 30 % dextran solution and the suspension was centrifuged at 4 °C for 25 min at 3000 g. The resulting vascular pellet was resuspended and triturated in WBB after which it was filtered through a 60 µm nylon mesh that allows to discard larger sized vessels. The capillary-enriched filtrate was centrifuged at 1000 g for 7 min at room temperature (RT) and the vascular pellet was then digested in WBB containing 10 mg/mL collagenase-dispase (Roche Diagnostics), 1,47 µg/mL Tosyl Lysin Chloromethyl Ketone (Sigma-Aldrich) and 10 µg/mL DNase I (Roche Diagnostics), for 33 min at 37 °C in a shaking water bath. Enzyme digestion was stopped by adding excess WBB and the vascular pellet, obtained by centrifuging at 1000 g for 7 min was triturated one more time. A final centrifugation at 1000 g (7 min, room temperature) resulted in a vascular pellet that was resuspended in DMEM containing glucose (1 g/L), Na^+-pyruvate (110 mg/L), glutamine (2 mM), gentamycin (50 µg/mL), BME vitamin solution (1%), BME amino acid solution (2%), basic fibroblast growth factor (bFGF, 1 ng/mL) and 20% newborn calf serum. The resulting cell suspension was plated onto matrigel-coated recipients and refreshed 24 h after seeding to remove red blood cells, cell debris and non-adherent cells. For some experiments, we used the capillary-enriched filtrate (see images Fig. 2B and D).

RBE4 (rat brain endothelial cells, obtained from Dr. F. Roux INSERM, Evry, France) were grown on collagen-coated recipients (rat tail collagen, Roche Diagnostics) and maintained in alpha-MEM/Ham's F10 (1:1) supplemented with 10% fetal bovine serum (FBS), glutamine (2 mM), geneticin (300 µg/mL) and basic fibroblast growth factor (bFGF, 1 ng/mL) at 37 °C and 5% CO_2.

HeLa cells stably transfected with Cx43 (HeLa-Cx43) were obtained from Prof. Dr. K. Willecke (Universität Bonn, Germany) (Elfgang et al., 1995). These cells were cultured in DMEM supplemented with 10% FBS, glutamine (2 mM) and puromycin (1 µg/ml), and maintained at

37°C and 10% CO_2. Untransfected HeLa wild type (WT) cells were grown in medium without puromycin. HeLa cells express TLR4 and IL6 receptors (Jiang et al., 2017; Miao et al., 2014).

Primary astrocytes were isolated from postnatal (P0-P1) cortices as previously described (Freitas-Andrade et al., 2019). Dissected cortices were triturated in DMEM and the resulting suspension was passed through a 70 µm cell filter before seeding into flasks. Cell culture medium (DMEM supplemented with 10% FBS, 10 units/mL penicillin, and 10 µg/mL streptomycin) was replaced 3 days after plating and every second day thereafter. After 7–8 days, astrocytes were harvested with trypsin-EDTA and frozen in freezing medium (90% FBS, and 10% DMSO). Frozen astrocytes were thawed and plated onto ploy-L-lysine (Sigma Aldrich) coated recipients. Cultures were maintained for 5–7 days prior to experiments.

Electrophysiological Recording

HeLa-Cx43 cells were seeded onto 13 mm diameter glass coverslips, RBE4 cells were seeded onto 13 mm diameter glass coverslips first coated with collagen and subsequently with Corning® Cell-Tak according to the supplier's instructions, primary astrocytes (55.000 cells) were plated onto 13 mm diameter glass coverslips coated with poly-L-lysine (Sigma Aldrich). RBE4 cells and primary astrocytes were plated at least 3 hrs prior to whole-cell patch-clamp recordings. This procedure yields round shaped and single cells in culture, making stable electrophysiological recordings possible. Experiments on HeLa cells were performed the next day. All experiments were performed on subconfluent (50%) cultures.

All recordings were performed in the presence of extracellular Ca^{2+} and Mg^{2+} and under conditions of K^+-channel blockade with Cs^+, Ba^{2+} and TEA^+. HeLa cells were bathed in a recording chamber filled with a modified Krebs-Ringer solution (pH 7.4) consisting of NaCl (150 mM), CsCl (6 mM), $MgCl_2$ (2 mM), $CaCl_2$ (2 mM), glucose (5 mM for HeLa cells and 10 mM for RBE4 cells and primary astrocytes), HEPES (5 mM), $BaCl_2$ (1 mM) and Pyruvate (2 mM) (Wang et al., 2012). The standard whole-cell recording pipette solution (pH 7.2) for all cells was composed of CsCl (130 mM), NaAsp (10 mM), $CaCl_2$ (0.26 mM), HEPES (5 mM), EGTA (2 mM), TEA-Cl (5 mM) and $MgCl_2$ (1 mM). For experiments on RBE4 cells and primary astrocytes, 5 mM MgATP was additionally added to the pipette solution. Free [Ca^{2+}] in the pipette solution (resembling the $[Ca^{2+}]_i$) was 50 nM, as calculated with Webmax Standard software

application (http://www.stanford.edu/~cpatton/webmaxcS.htm). Other estimations of attained $[Ca^{2+}]_i$ were also calculated using this software. For study of LPS- or IL6-dependent activation of Cx43 hemichannels in respectively, RBE4 cells or primary astrocytes, 2 mM EGTA was changed by 0.1 mM EGTA and no Ca^{2+} was added in the pipette solution. For intracellular Ca^{2+} chelating experiments, EGTA in the pipette solution was replaced by 10 mM BAPTA. For experiments with Gap19 (KQIEIKKFK, no Tat conjugation), 100 µM peptide was added to the standard pipette solution. Cells were exposed to LPS (1 µg/mL), IL6 (100 ng/mL) and Gap27 (300 µM) via a fast local perfusion system. For study of IL6-dependent activation of Cx43 hemichannels in RBE4 cells, cells were pre-incubated with 200 µM Gap27 for 30 minutes. Different conditions were tested in random order to minimize time-dependent changes following plating. An EPC 7 PLUS patch-clamp amplifier (HEKA Elektronik, Lambrecht/ Pfalz, Germany) was used to perform single channel recordings. Data were acquired at 6 kHz using a NI USB-6221 data acquisition device from National Instruments (Austin, TX, USA) and WinWCP acquisition software (designed by Dr. J. Dempster; University of Strathclyde, UK). All currents in whole-cell configuration were filtered at 1 kHz (7-pole Besselfilter). For single channel analysis, holding currents were subtracted from the recorded current traces, giving traces that only contained unitary current events. Unitary conductance was calculated from the elementary current transitions Δi as: $\gamma = \Delta i/V_m$. From these data, we constructed all-point conductance histograms that displayed one or more Gaussian distributions. These were fit by a probability density function assuming independent channel opening (Ramanan and Brink, 1993; Wang et al., 2001; Wang et al., 2012; Wang et al., 2013). Channel activity was quantified from the charge transfer Q_m associated with unitary currents; this was done by integrating the unitary current traces (i.e., a function of time) over the duration of the voltage step as: $Q_m = \int i dt$. Additionally, unitary events in the tail current were counted manually.

Calcium imaging

RBE4 cells were seeded onto 18 mm diameter glass coverslips coated with collagen and Corning® Cell-Tak, according to the manufacturer's instructions and primary astrocytes were seeded onto these same coverslips coated with poly-L-lysine (Sigma Aldrich). No coating was necessary for HeLa-Cx43 cells. Experiments were all performed at 50-70% confluency. Cells were loaded with a mixture of 5-10 µM Fluo3- AM and 0.01 % pluronic acid in HBSS during 1

h at room temperature (RT). For endothelial cells, HBSS-Hepes additionally contained 1 mM probenecid. After loading, cells were washed and left for an additional 30 min at RT in HBSS-Hepes to allow for de-esterification. Cells were then transferred to an inverted epifluorescence microscope (Eclipse TE 300, Nikon Belux), equipped with a superfusion system that allowed changing the bath solution within ~1 min (bath volume ~300 µL). Superfusion was switched off during the registration of oscillatory activity. Images were taken every second with a x 40 water immersion objective (NA 0.8) and an electron multiplying CCD camera (Quantem 512SC, Photometrics, Tucson, AZ, USA). We used a Lambda DG-4 filter switch (Sutter Instrument Company, Novato, CA, USA) to deliver excitation at 482 nm and captured emitted light via a 505 nm long-pass dichroic mirror and a 535 nm bandpass-filter (35 nm bandwidth). Recordings and analysis were done with custom-developed QuantEMframes and Fluoframes software written in Microsoft Visual C++ 6.0. Ca^{2+} oscillations were counted in a 10 min observation period and were defined as at least two transient Ca^{2+} changes following the initial Ca^{2+} transient in individual cells, minimally 5 % above baseline Fluo3-fluorescence.

Immunohistochemistry

Immunohistochemical analysis of protein expression was performed on coronal mouse brain cryosections or freshly isolated capillaries (see above under Cell isolation and cell culture studies). Samples were fixed in 4 % paraformaldehyde (PFA) and permeabilized with 0.1 % Triton X-100 (TX100). Samples were then pre-incubated for 1 h in 10 % normal goat serum (NGS) and 1 % BSA before addition of primary antibodies diluted in 1 % NGS - 1 % BSA solution. After washing, secondary antibody conjugated to different Alexa Fluor Dyes (Molecular Probes, Invitrogen) and diluted in 1 % NGS - 1 % BSA solution was incubated for 2 h at RT. DAPI (4′,6-Diamidine-2′-phenylindole dihydrochloride) was used as a nuclear stain. For capillary segments, all incubation and washing steps were performed on a rotator. Samples were mounted in ProLong Gold Antifade mounting medium (Invitrogen). Primary antibodies were rat anti-CD31 (Invitrogen/BD Biosciences), rabbit anti-AQP4 (Merck Millipore), rabbit-GFAP (Abcam), GFAP-Cy3 (Invitrogen), rabbit anti-Cx43 (Sigma-Aldrich) and mouse anti-Cx43 (Merck Millipore/BD Biosciences).

The samples were visualized by confocal microscopy (Leica TCS SP8 X; 63x water objective).

For evaluation of the connexin expression in BBB capillaries and perivascular endfeet, we used the ImageJ coloc plugin to count colocalized pixels. Counts were expressed relative to the number of CD31-positive endothelial cell pixels or AQP4-positive astrocytic endfeet pixels.

Immunohistochemical analysis of GFAP fluorescence was quantified from coronal brain cryosections and expressed relative to the signal observed in non-treated control animals (number of animals as in the treated group). Fluorescence was visualized by confocal microscopy (Leica TCS SP8 X; 63x water objective) and quantified in the somatosensory cortex.

Gel electrophoresis and western blotting

Total cell lysates were extracted with radioimmunoprecipitation assay (RIPA) buffer (25 mM Tris, 50 mM NaCl, 0.5% Nonidet P-40, 0.5% deoxycholate, 0.1% SDS, 1 mM DTT, 0.055 g/ml β-glycerol phosphate, 20 µl/ml phosphatase and protease inhibitor cocktail, and 20 µl/ml mini EDTA-free protease inhibitor mix). Protein concentration was determined using the Biorad DC protein assay kit (BioRad, Nazareth, Belgium) and absorbance was measured with a 590 nm long-pass filter. Whole tissue lysates were extracted by homogenization and sonication in RIPA buffer. Cell lysates and plasma samples were separated by electrophoresis over a 4-12 % SDS-polyacrylamide gel and transferred to a nitrocellulose membrane (Amersham, Buckinghamshire, UK). Membranes were subsequently blocked with TBS containing 5 % non-fat milk and 0.1 % Tween20. Following blocking, blots were probed with rabbit anti-Cx43 antibody (Sigma-Aldrich) or rabbit anti-S100β (Abcam, Cambridge, UK). Membranes were subsequently incubated with an alkaline phosphatase-conjugated goat anti-rabbit IgG antibody (Sigma-Aldrich) and detection was done using the nitro-blue-tetrazolium/5-bromo-4-chloro-3-indolyl-phosphate reagent (NBT/BCIP kit, Zymed, Invitrogen). Alternatively, membranes were incubated with HRP-conjugated goat anti-rabbit IgG (Santa Cruz) and detection was done using SuperSignal™ West Femto Maximum Sensitivity Substrate (Thermo Fisher Scientific). Total protein stains by SYPRO® Ruby (Molecular probes) prior to antibody incubation, or detection with rabbit anti-β-tubulin

antibody (Abcam) were used as loading controls. Quantification was done by drawing a rectangular window around the concerned protein band and determining the signal intensity using ImageJ. Background correction was done by the same procedure applied to nitrocellulose membranes where protein was absent.

Figures

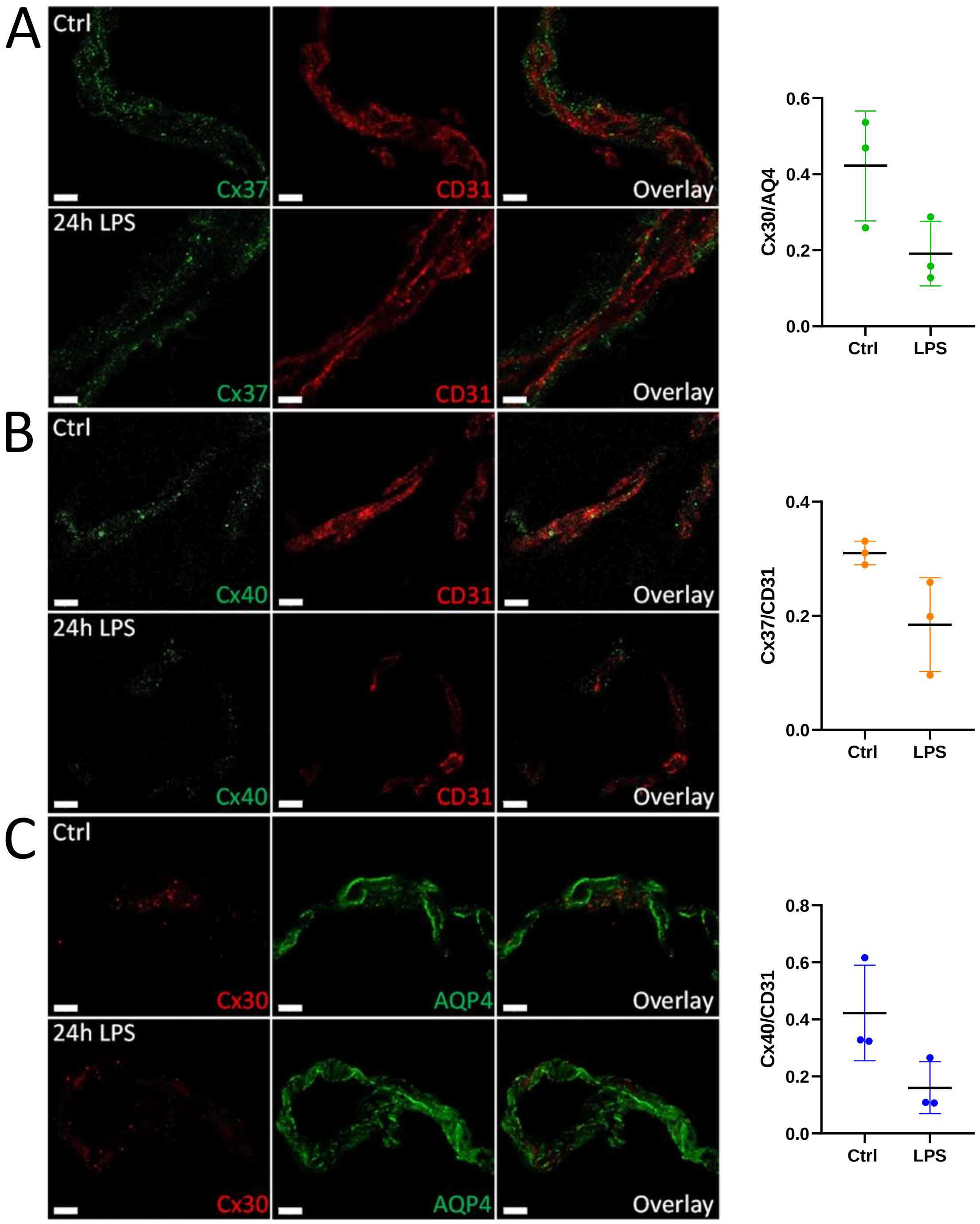

Figure S1. Effect of LPS treatment on the expression of astrocytic Cx30 and endothelial Cx37 and Cx40 as determined in brain capillaries freshly isolated from mice. The expression levels tended to be decreased by LPS but this did not attain statistical significancy (unpaired t-test; n = 3). Antibodies were as follows: rabbit anti-Cx30 (1/500; Thermofisher) and chicken anti-AQP4 (1/1000; Synaptic systems); rabbit anti-Cx37 (1/500; Thermofisher), rabbit anti-Cx40 (1/200; Thermofisher) and rat anti-CD31 (1/100; Becton Dickinson).

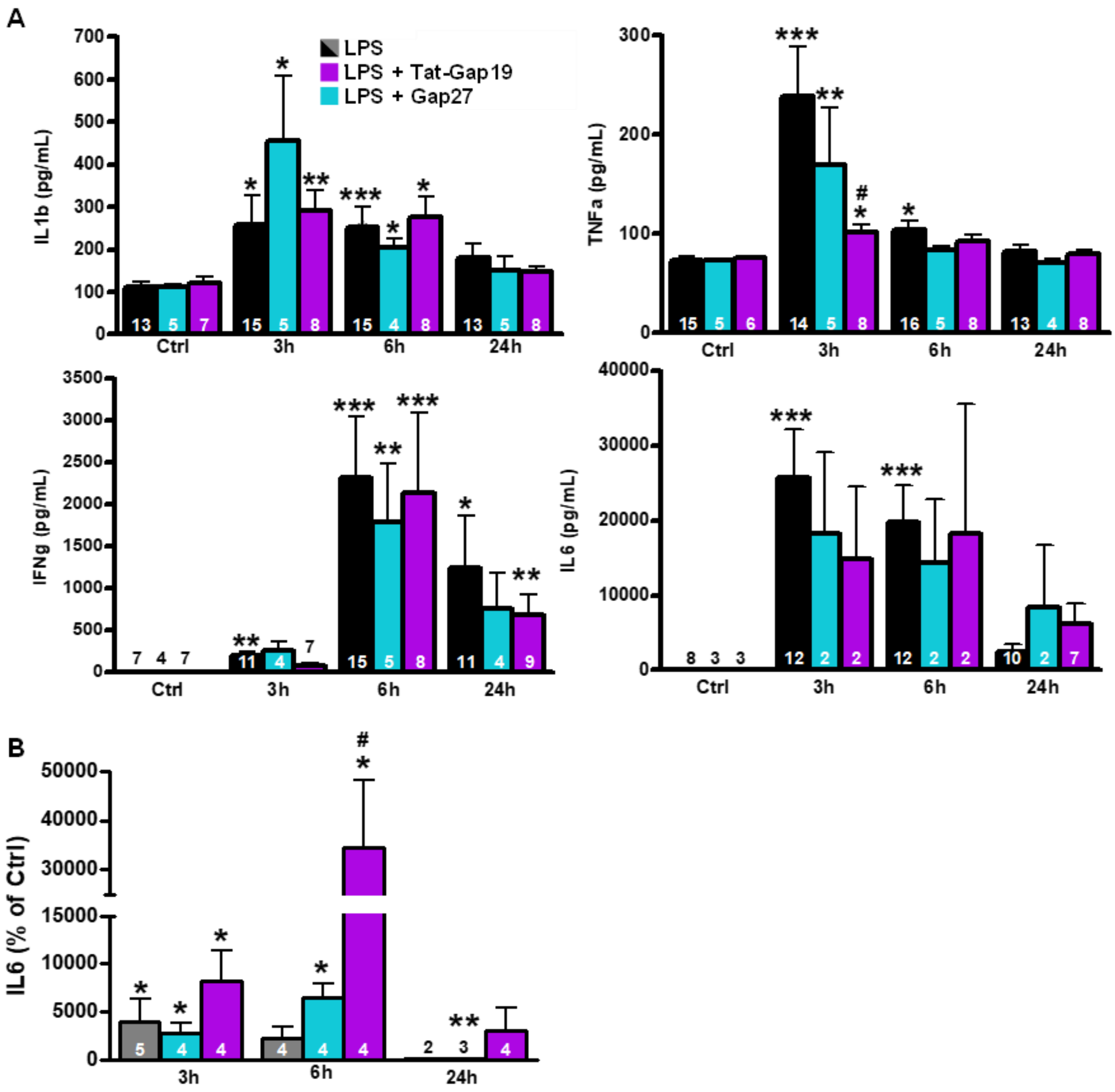

Figure S2. Effect of Gap27 and Tat-Gap19 on plasma and brain levels of IL1β, TNFα, IFNγ and IL6 following LPS stimulation. Plasma (A) and brain (B) levels of the tested cytokines after LPS stimulation were not significantly affected by Gap27. Tat-Gap19 significantly decreased TNFα in the blood and increased IL6 in the brain. Stars indicate significant difference with Ctrl (saline

IP) while number signs indicate significant difference with LPS; one-way ANOVA, Bonferroni test except for IL1β & TNFα where non-parametric Kruskal-Wallis testing was used.

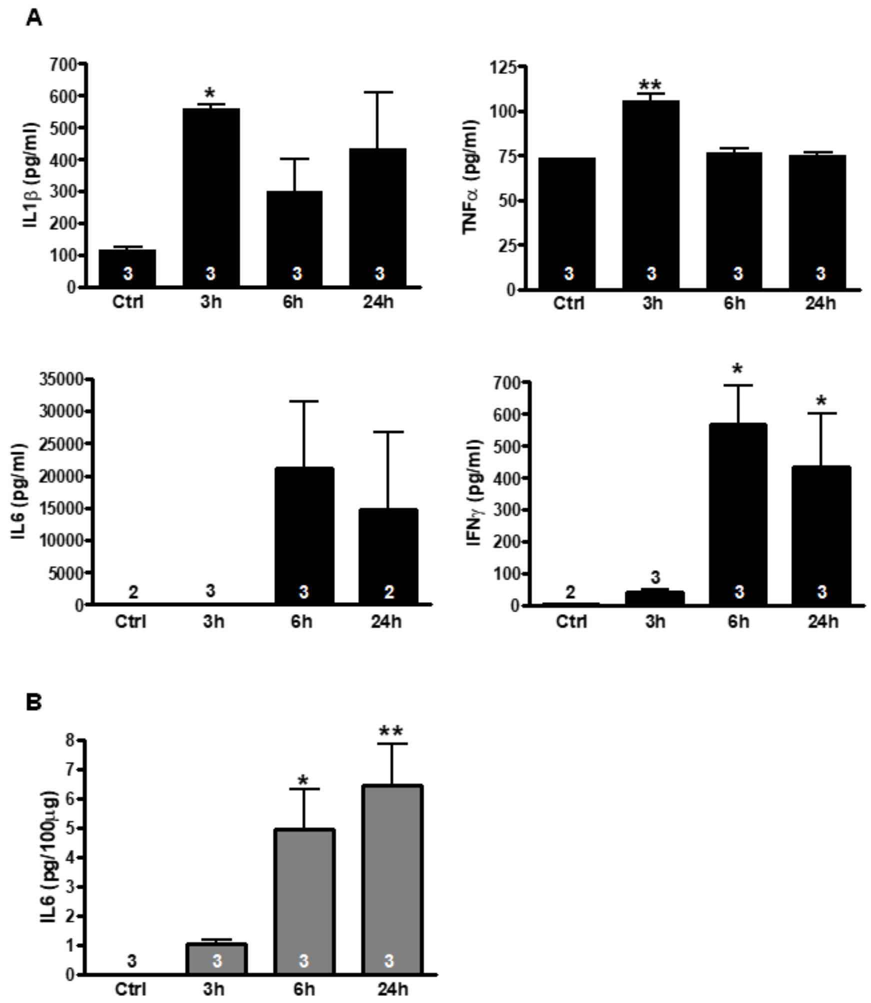

Figure S3. Effect of BAPTA-AM treatment on plasma and brain levels of IL1β, TNFα, IFNγ and IL6 following LPS stimulation. Plasma (A) and brain (B) levels of the tested cytokines after LPS stimulation and IV BAPTA-AM treatment. Comparison to LPS without BAPTA-AM (Fig. 1D-E) indicated no effects on IL1β, TNFα and IFNγ while IL6 elevation appeared delayed in plasma and brain (plasma IL6 in Ctrl and at 3 h were undetectable). Star symbols indicate significant difference with Ctrl (saline IP; one-way ANOVA, Dunnett test).

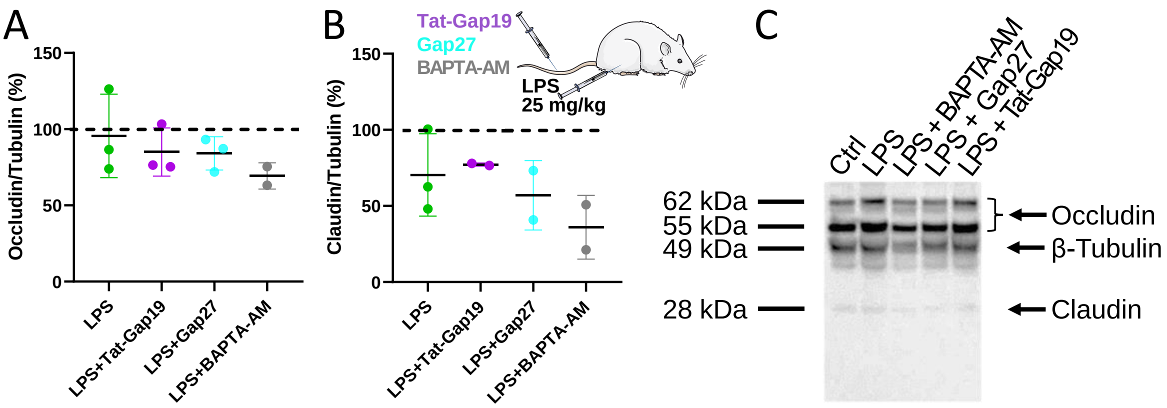

Figure S4. Effect of LPS and LPS combined with peptide/BAPTA-AM treatments on occludin and claudin expression levels in brain endothelial cells isolated from mice. Antibodies were as follows: rabbit anti-β-tubulin (1/1000; Abcam); rabbit anti-claudin 5 (1/500; Santa Cruz) and rabbit anti-occludin (1/1000; Thermofisher).

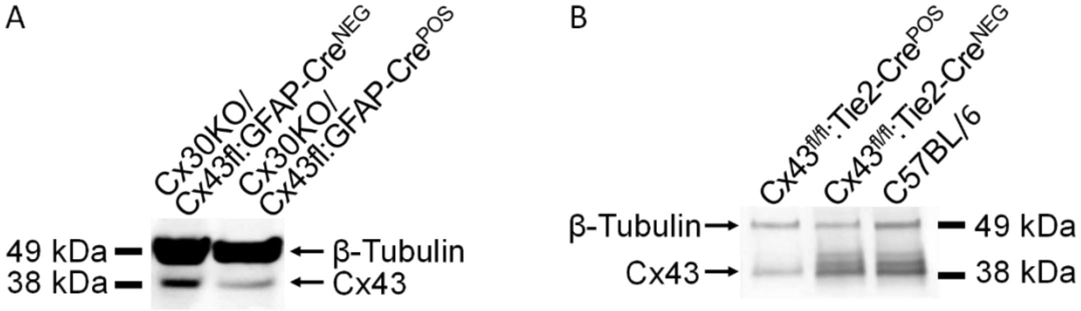

Figure S5. Representative Western blottings illustrating the efficiency of Cx43 KO in astrocytes (A) and endothelial cells (B).

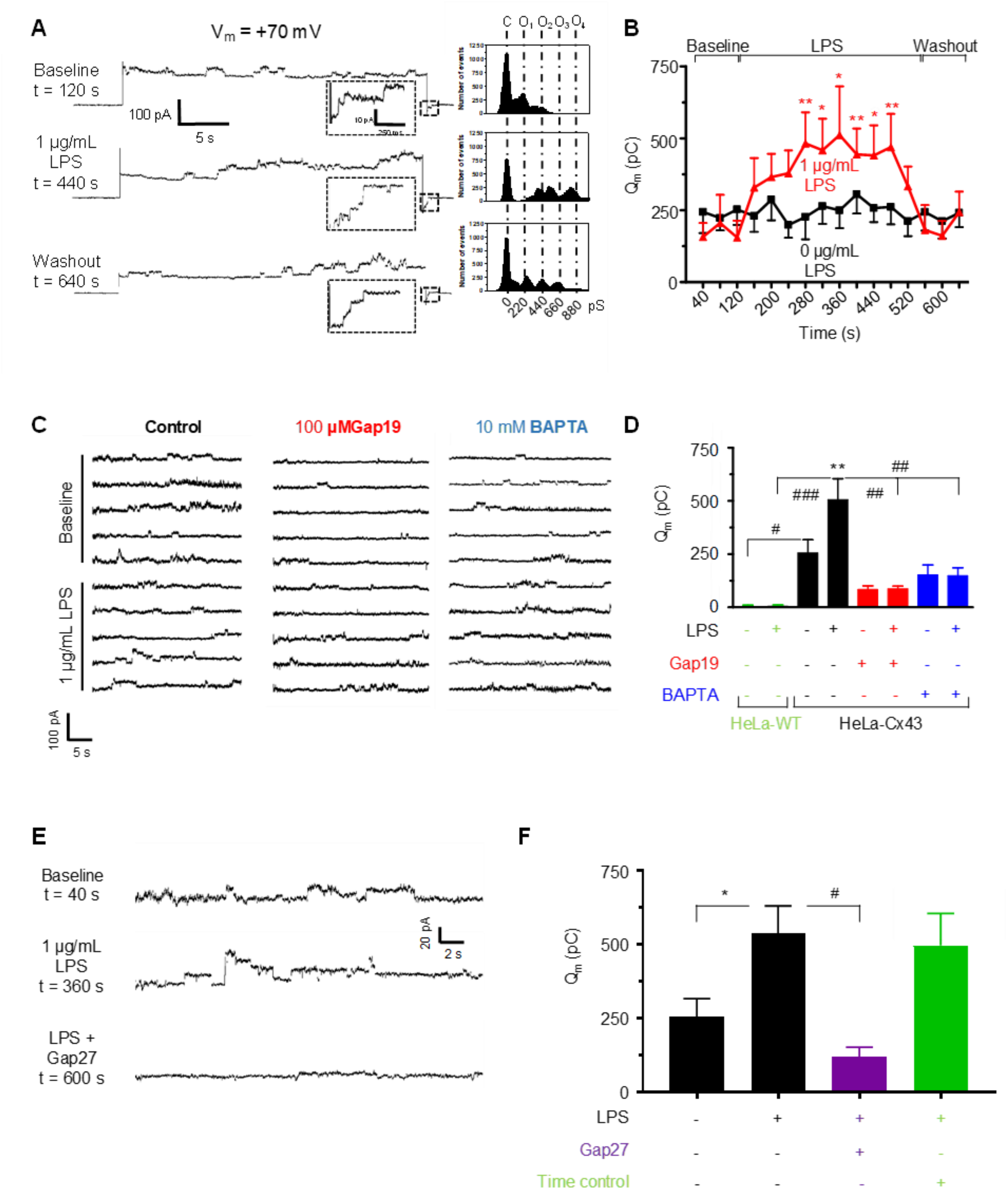

Figure S6. LPS enhances Ca^{2+}-dependent Cx43 hemichannel opening in HeLa cells stably transfected with Cx43. A. Example traces and matching all-point histograms depicting representative voltage-induced (+70 mV, 30 s) Cx43 hemichannel unitary currents recorded in HeLa-Cx43 cells before (baseline), during and after washout of LPS (1 µg/mL) applied via fast local superfusion. Insets (dashed boxes) show typical staircase-like channel closing events in the tail currents at repolarization. **B.** Q_m summary data for repeated current measurements

for LPS (1 µg/mL, red trace) and control (0 µg/mL LPS, black trace) (n_{cells} = 9 per concentration; 5 independent experiments). Red stars compare to 40 s point (repeated measures ANOVA, Dunnett test). Average Q_m during LPS (160-520 s) was significantly above control without LPS ($p<0.05$; two-sample t-test). **C.** Representative current traces illustrating the effect of Gap19 and BAPTA applied via the patch pipette. **D.** Q_m summary data of experiments as in panel C (n_{cells} = 9-17 per condition; 5 independent experiments). Stars compare LPS versus control without LPS; number signs compare between conditions indicated by the lines (one-way ANOVA, Bonferroni test). **E.** Representative current traces illustrating the effect of Gap27 (3 min wash-in). **F.** Q_m summary data of experiments as shown in panel E (n_{cells} = 9 per condition; 5 independent experiments). Star compares LPS versus control without LPS; number sign compares Gap27 effect to LPS-only (one-way ANOVA, Dunnett test).

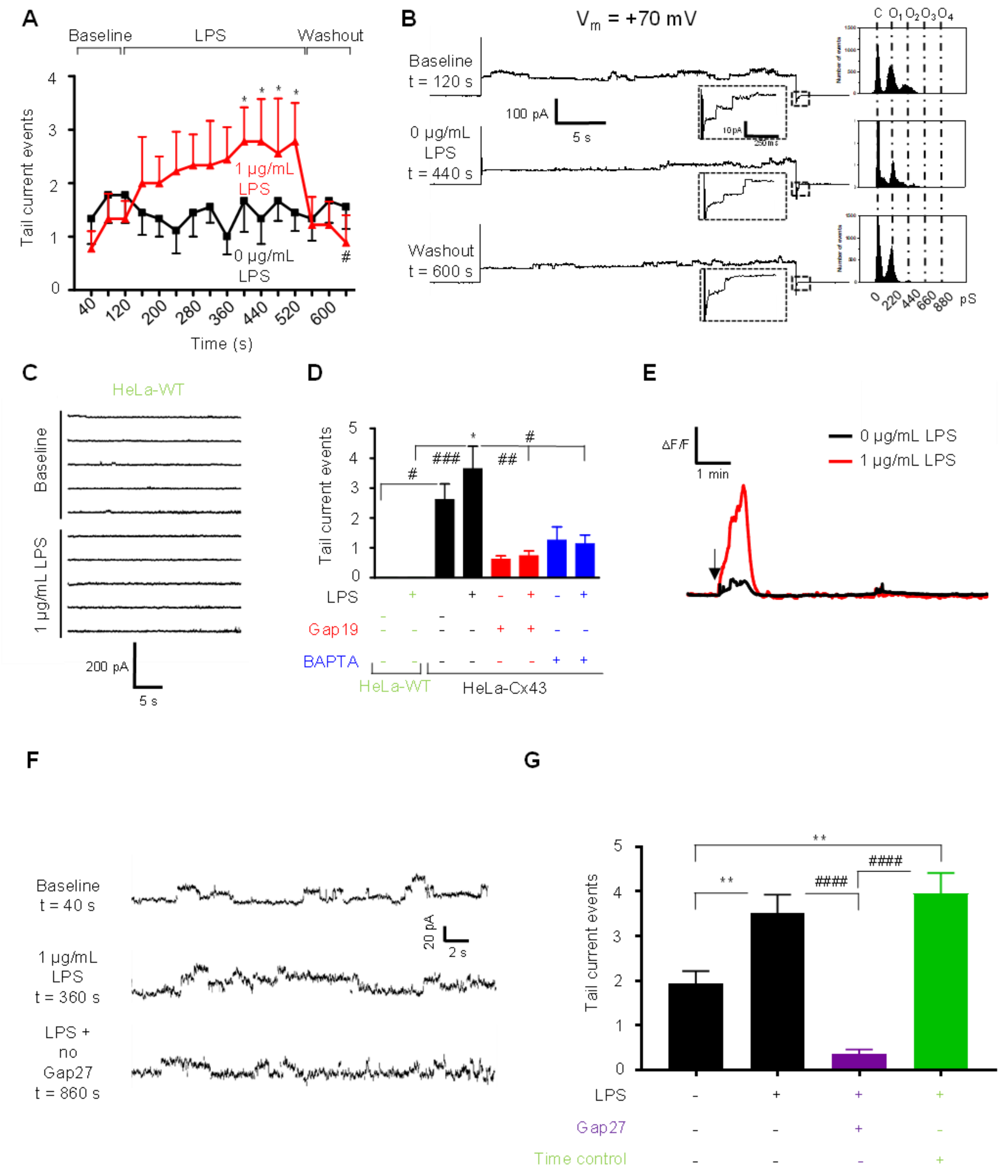

Figure S7. LPS enhances Ca^{2+}-dependent Cx43 hemichannel opening in HeLa cells stably transfected with Cx43 - tail current hemichannel closing analysis. A. Tail current hemichannel closing analysis of experimental data of Fig. S6A-B (staircase-like channel closings). Graph illustrates summary data for hemichannel closing event counts during baseline, LPS (1 µg/mL) and washout (red trace; black trace shows corresponding event counts in the absence of LPS) (n_{cells} = 9 per concentration; 5 independent experiments). Red stars compare to 40 s point (repeated measures ANOVA, Dunnett test). **B.** Negative control

experiment for Fig. S6A. Example traces and matching all-point histograms for voltage-induced Cx43 hemichannel unitary current activity recorded in HeLa-Cx43 cells during baseline, 0 µg/mL LPS exposure and washout. Insets illustrate staircase-like channel closing events in the tail currents of the corresponding traces following repolarization. **C.** Negative control experiment of Fig. S6C-D recorded in HeLa-WT cells in baseline and following stimulation with 1 µg/mL LPS. **D.** Summary bar chart of hemichannel closing event analysis of the experimental data from Fig. S6D (n_{cells} = 9-17 per condition; 5 independent experiments). Stars compare LPS versus control without LPS; number signs compare between LPS and conditions indicated by the lines (one-way ANOVA, Bonferroni test). **E.** Example trace demonstrating an LPS-induced Ca^{2+} transient in HeLa-Cx43 cells (1 µg/mL LPS); vehicle-only superfusion (0 µg/mL LPS) had no effect. The black arrow marks introduction of LPS or vehicle. **F.** Representative current traces illustrating the effect of Gap27. **G.** Summary data of hemichannel closing event analysis experiments as shown in panel F (n_{cells} = 9 per condition; 5 independent experiments). Stars compare LPS versus control without LPS; number signs compare Gap27 effect to LPS-only (one-way ANOVA, Dunnett test).

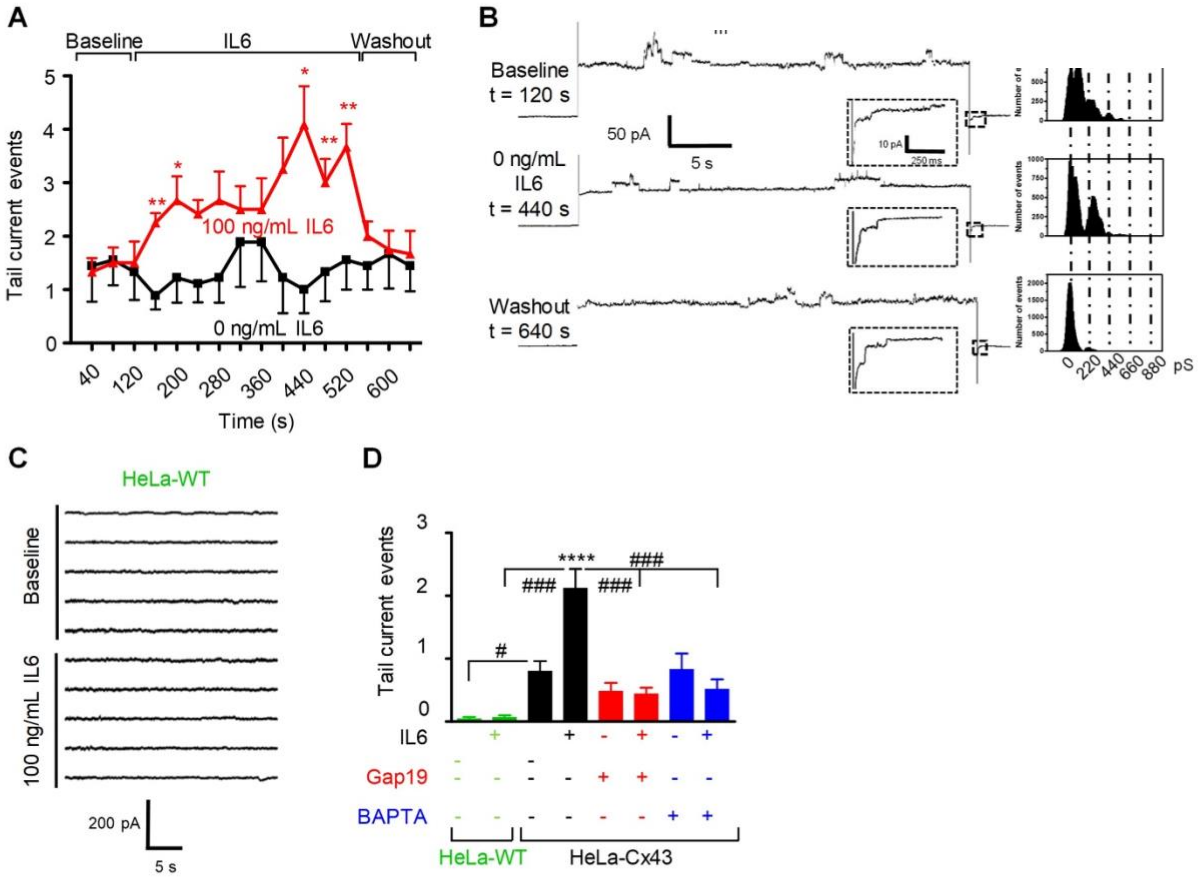

Figure S8. IL6 enhances opening of Cx43 hemichannels in a Ca^{2+}-dependent manner - tail current hemichannel closing analysis. A. Tail current hemichannel closing analysis of experimental data in HeLa-Cx43 cells as presented in Fig. 10A-B. Summary data for hemichannel closing event counts during baseline, IL6 (100 ng/mL) and washout (red trace; black trace shows corresponding event counts in the absence of IL6) (n_{cells} = 9-12 per concentration; 6 independent experiments). Red stars compare to 40 s point (repeated measures ANOVA, Dunnett test). **B.** Negative control experiment for Fig. 10A. Example traces and matching all-point histograms for voltage-induced Cx43 hemichannel unitary current activity recorded in HeLa-Cx43 cells during baseline, 0 ng/mL IL6 exposure and washout. Insets show channel closing events in the tail currents of the corresponding traces following repolarization. **C.** Negative control experiment of Fig. 10C-D recorded in HeLa-WT cells in baseline and following stimulation with 100 ng/mL IL6. **D.** Summary bar chart of hemichannel closing event analysis of the experimental data from Fig. 10C-D (n_{cells} = 9-11 per condition; 6 independent experiments). Stars compare IL6 versus control without IL6; number signs compare between conditions indicated by the lines (one-way ANOVA, Bonferroni test).

References

Dantzer, R., J.C. O'Connor, G.G. Freund, R.W. Johnson, and K.W. Kelley. 2008. From inflammation to sickness and depression: when the immune system subjugates the brain. *Nature reviews. Neuroscience*. 9:46-56.

Elfgang, C., R. Eckert, H. Lichtenberg-Frate, A. Butterweck, O. Traub, R.A. Klein, D.F. Hulser, and K. Willecke. 1995. Specific permeability and selective formation of gap junction channels in connexin-transfected HeLa cells. *The Journal of cell biology*. 129:805-817.

Freitas-Andrade, M., N. Wang, J.F. Bechberger, M. De Bock, P.D. Lampe, L. Leybaert, and C.C. Naus. 2019. Targeting MAPK phosphorylation of Connexin43 provides neuroprotection in stroke. *The Journal of experimental medicine*. 216:916-935.

Jiang, N., F. Xie, Q. Guo, M.Q. Li, J. Xiao, and L. Sui. 2017. Toll-like receptor 4 promotes proliferation and apoptosis resistance in human papillomavirus-related cervical cancer cells through the Toll-like receptor 4/nuclear factor-kappaB pathway. *Tumour biology : the journal of the International Society for Oncodevelopmental Biology and Medicine*. 39:1010428317710586.

Lutz, S.E., Y. Zhao, M. Gulinello, S.C. Lee, C.S. Raine, and C.F. Brosnan. 2009. Deletion of astrocyte connexins 43 and 30 leads to a dysmyelinating phenotype and hippocampal CA1 vacuolation. *The Journal of neuroscience : the official journal of the Society for Neuroscience*. 29:7743-7752.

Miao, J.W., L.J. Liu, and J. Huang. 2014. Interleukin-6-induced epithelial-mesenchymal transition through signal transducer and activator of transcription 3 in human cervical carcinoma. *International journal of oncology*. 45:165-176.

Ramanan, S.V., and P.R. Brink. 1993. Multichannel recordings from membranes which contain gap junctions. II. Substates and conductance shifts. *Biophysical journal*. 65:1387-1395.

Teubner, B., V. Michel, J. Pesch, J. Lautermann, M. Cohen-Salmon, G. Sohl, K. Jahnke, E. Winterhager, C. Herberhold, J.P. Hardelin, C. Petit, and K. Willecke. 2003. Connexin30 (Gjb6)-deficiency causes severe hearing impairment and lack of endocochlear potential. *Hum Mol Genet*. 12:13-21.

Theis, M., R. Jauch, L. Zhuo, D. Speidel, A. Wallraff, B. Doring, C. Frisch, G. Sohl, B. Teubner, C. Euwens, J. Huston, C. Steinhauser, A. Messing, U. Heinemann, and K. Willecke. 2003. Accelerated hippocampal spreading depression and enhanced locomotory activity in mice with astrocyte-directed inactivation of connexin43. *The Journal of neuroscience : the official journal of the Society for Neuroscience*. 23:766-776.

Wallraff, A., R. Kohling, U. Heinemann, M. Theis, K. Willecke, and C. Steinhauser. 2006. The impact of astrocytic gap junctional coupling on potassium buffering in the hippocampus. *The Journal of neuroscience : the official journal of the Society for Neuroscience*. 26:5438-5447.

Wang, H.Z., N. Day, M. Valcic, K. Hsieh, S. Serels, P.R. Brink, and G.J. Christ. 2001. Intercellular communication in cultured human vascular smooth muscle cells. *American journal of physiology. Cell physiology*. 281:C75-88.

Wang, N., M. De Bock, G. Antoons, A.K. Gadicherla, M. Bol, E. Decrock, W.H. Evans, K.R. Sipido, F.F. Bukauskas, and L. Leybaert. 2012. Connexin mimetic peptides inhibit Cx43 hemichannel opening triggered by voltage and intracellular Ca2+ elevation. *Basic research in cardiology*. 107:304.

Wang, N., E. De Vuyst, R. Ponsaerts, K. Boengler, N. Palacios-Prado, J. Wauman, C.P. Lai, M. De Bock, E. Decrock, M. Bol, M. Vinken, V. Rogiers, J. Tavernier, W.H. Evans, C.C. Naus, F.F. Bukauskas, K.R. Sipido, G. Heusch, R. Schulz, G. Bultynck, and L. Leybaert. 2013. Selective inhibition of Cx43 hemichannels by Gap19 and its impact on myocardial ischemia/reperfusion injury. *Basic research in cardiology*. 108:309.
